# Comparative Analysis of Relative Ligand Binding Free Energy Simulation Methods: Amber-TI, GROMACS-NETI, OpenMM-FEP, and BLaDE-MSLD

**DOI:** 10.1021/acs.jctc.6c01744

**Published:** 2026-10-09

**Authors:** Hajin Lee, Injung Kim, Sungjun Kim, Minsik Bae, Bomi Jeong, Seonghoon Kim, Sunhwan Jo, Jumin Lee, Wonpil Im

**Affiliations:** † MolCube Inc., Seoul 06704, Republic of Korea; ‡ Department of Biological Sciences, 1687 Lehigh University, Bethlehem, Pennsylvania 18015, United States

## Abstract

Structure-based drug design has become increasingly important in the pharmaceutical industry for accelerating the discovery of effective drug candidates. In particular, ligand binding free energy serves as a critical metric for predicting drug efficacy during the key stages of hit discovery and lead optimization. Continuous progress has been made in the prediction of ligand binding free energies, but direct comparisons of different methods using the same force field remain challenging due to their unique implementations in different simulation engines. In this study, we present a direct comparison of four popular methodologies (Amber-TI, GROMACS-NETI, OpenMM-FEP, and BLaDE-MSLD) for calculating relative binding free energies (ΔΔ*G*_bind_) with the same Amber protein and ligand force fields using *MolCube Alchemical Free Energy Simulator* (*MolCube-AFES*), which provides an input generation workflow to support ΔΔ*G*_bind_ calculations of all four methods. We used 80 alchemical transformations (among the JACS benchmark set by Wang et al.) and two additional applications to compare the predicted ΔΔ*G*_bind_ from the four methods against experimental measurements. All four methods reproduced experimentally observed trends, with most transformations within ±2 kcal/mol from experiments, and showed broadly comparable accuracy with no statistically significant performance differences across the benchmark data set. These results demonstrate that *MolCube-AFES* enables controlled, cross-platform benchmarking and show that all four different alchemical free energy methods deliver statistically equivalent accuracy, with method selection guided by workflow requirements such as throughput, portability, and perturbation network design rather than expected differences in performance.

## Graphical abstract

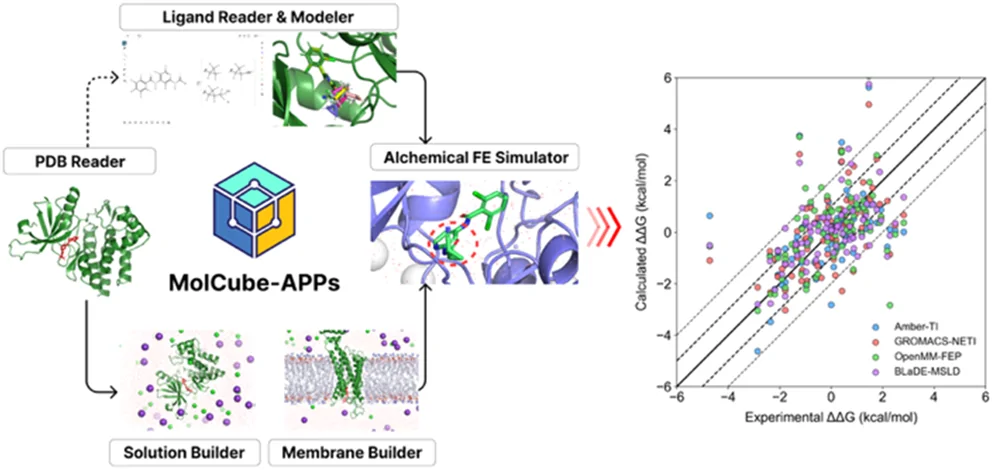

## 1 Introduction

Free energy calculations are essential tools in molecular simulations, providing quantitative insights into thermodynamic properties such as binding affinities, solvation free energies, and conformational stability. Particularly, alchemical free energy simulation (AFES) methods have become important tools in structure-based drug design, where accurate estimation of relative or absolute binding free energies^1–4^ can guide hit discovery and lead optimization and thus reduce experimental overhead.

Multiple theoretical frameworks have been developed for performing free energy calculations. Thermodynamic integration (TI)^5^ remains one of the most widely adopted equilibrium-based methods, while nonequilibrium TI (NETI),^6^ grounded in Jarzynski’s equality, has gained popularity for its computational efficiency. Free energy perturbation (FEP),^7^ which originated from Zwanzig’s exponential averaging formula as a historical and theoretical foundation, has long been a foundational approach for calculating relative free energies, particularly in alchemical transformations. More recently, multisite lambda dynamics (MSLD)^8^ has emerged as a promising approach for handling complex perturbation networks such as ligand series with multiple substitution sites.

In accordance with these theoretical frameworks, several software packages have implemented optimized engines for AFES. For example, Amber^9^ provides comprehensive support for the TI-based free energy calculation. GROMACS,^10^ in combination with external libraries such as *pmx*,^11,12^ enables the execution of NETI-based protocols. BLaDE,^8^ a GPU-accelerated platform, offers a dedicated implementation of MSLD, while Schrödinger’s *FEP+* ^13^ and OpenMM-powered *OpenFE* ^14^ provide widely used implementations of alchemical free energy method for efficient relative free energy predictions.

While each method and simulation engine combination (Amber-TI, GROMACS-NETI, OpenMM-FEP, and BLaDE-MSLD) has been independently benchmarked,^8,14–17^ a systematic side-by-side comparison using the same molecular system and force field has not been yet reported due to unique implementations of these methods into different simulation engines, as well as the intended force field support. Addressing this challenge is further complicated by the lack of a unified tool to prepare the system and input files for all four methods in the same conditions. To overcome this limitation, we have developed *MolCube Alchemical Free Energy Simulator* (*MolCube-AFES*), an advanced module integrated into *MolCube-APPs* (https://apps.molcube.com), to streamline and standardize input generation for AFES across platforms. This study leverages *MolCube-AFES* to perform a comprehensive comparison of the four approaches on representative molecular systems, evaluating their accuracy, convergence behavior, computational efficiency, and practical usability. Our findings aim to provide valuable insights into the strengths, limitations, and optimal use cases of each method.

## 2 Methods

### 2.1 System Setup and Benchmark Targets

The protein–ligand complex structures used in this study were selected based on their relevance to the experimental ligand series. Specifically, we prioritized structures either directly derived from studies reporting binding affinities for the ligands of interest or those containing cocrystallized ligands with high structural similarity to the target compounds. Our study focused on the eight protein targets originally introduced by Wang et al.,^13^ the so-called JACS benchmark set widely used to assess AFES performance. To broaden the comparative scope, we additionally included a glucocorticoid receptor (GR) system described by Weinstein et al.^18^ and an HDAC6 inhibitor system from Wang et al.^19^ For the JACS benchmark targets, the initial protein–ligand complexes were obtained from experimentally determined structures deposited in the Protein Data Bank (PDB),^20^ with the following accession codes: BACE (4DJW),^21^ Tyk2 (4GIH),^22^ CDK2 (1H1Q),^23^ MCL1 (4HW3),^24^ JNK1 (2GMX),^25^ p38 (3FLY),^26^ thrombin (2ZFF),^26^ and PTP1B (2QBS).^27^ Because no cocrystal structure is available for the GR system, the protein–ligand complex was generated using Boltz-2,^28^ referencing PDB ID 3BQD for the binding-site conformation. For the HDAC6 inhibitor system, we used the docking model reported by Wang et al.,^19^ derived from the PDB ID 5EDU receptor structure, which served as our structural template in the absence of an experimentally determined complex.

To systematically and efficiently perform large-scale ΔΔ*G*_bind_ calculations, we selected 10 transformations from each protein system in the JACS benchmark set (Table S1), ensuring that their ΔΔ*G*_bind_ values span a relatively broad range based on available experimental data. Following the transformation-selection strategy described by Han et al.,^29^ this panel was constructed to capture the chemically diverse modifications typically explored during lead optimization in real-world drug discovery campaigns, including small substituent exchanges, ring additions or deletions, functional-group interconversions, and stereochemical changes. By incorporating both minor perturbations (e.g., hydrogen-to-halogen substitutions) and more substantial structural alterations, we aimed to assess the performance and generalizability of *MolCube-AFES* across a wide spectrum of chemically meaningful transformations.

The preparation of all simulation systems was streamlined through *MolCube-APPs* (Figure 1). Protein structures were prepared using the automated protocol in *PDB Reader*, which reconstructs any necessary missing residues, assigns protonation states assuming a pH of 7, and retains relevant crystallographic water molecules. Each system was solvated using *Solution Builder* in a cubic box with a 10 Å buffer of explicit water molecules and neutralized with 0.15 M KCl. The force field parameters were assigned using Amber ff19SB for proteins,^30^ OPC^31^ for water molecules in Amber-TI, GROMACS-NETI, and OpenMM-FEP and TIP3P^32^ for BLaDE-MSLD, and GAFF2.20 ^33^ with AM1-BCC charges^34^ for ligands. Note that we employed the TIP3P water model for BLaDE-MSLD due to the technical complexity of integrating the virtual site of OPC water into BLaDE, and the previous study by Han et al. shows minor influences of water models on ΔΔ*G*_bind_.^29^

**Figure 1:**
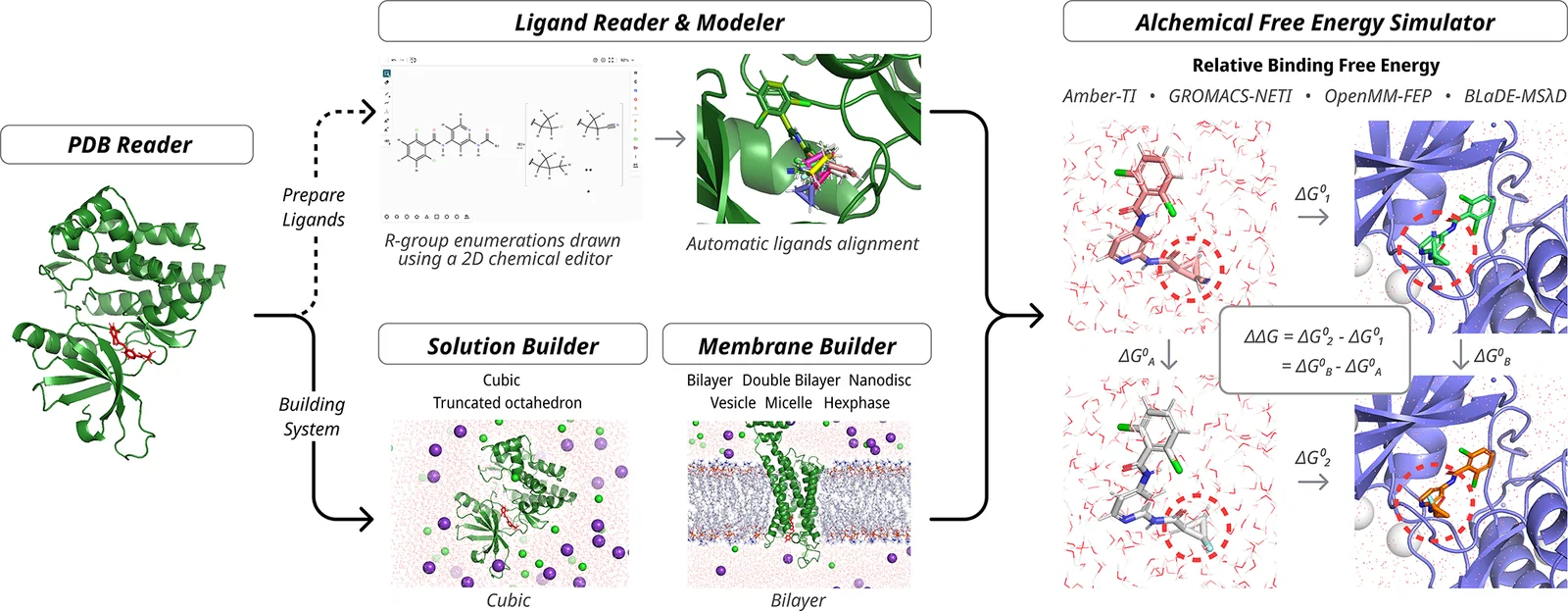
Schematic overview of the alchemical free energy simulation workflow implemented in *MolCube-APPs*.

A series of congeneric ligands for each system were prepared using *Ligand Reader & Modeler (LRM)*, which (1) reads ligands from various sources, including uploaded SDF files or R-group enumerations drawn using a 2D chemical editor, (2) aligns them to the reference ligand from the corresponding *PDB Reader* project, and (3) assigns appropriate force field parameters. The procedure employs a maximum common substructure (MCS) algorithm^35^ to identify the shared atomic core, from which coordinates of the common atoms are directly transferred. A constrained conformation search is subsequently conducted for the remaining atoms, followed by a short energy minimization to yield a reasonable initial conformation. *LRM* automatically issues warnings for potential atomic overlaps, and users may manually adjust conformations if necessary. In this study, all systems were generated automatically, as potential overlaps are generally resolved during the minimization phase of the simulation.

*MolCube-AFES* then takes the prepared ligand sets and the solvated (or membrane) system to generate input files for Amber-TI, GROMACS-NETI, OpenMM-FEP, and BLaDE-MSLD relative binding free energy simulation, automating key steps in the preparation of perturbation networks and topologies. Figure 2A,B shows screenshots of the *MolCube-AFES* user interface, illustrating a typical workflow for constructing AFES calculation systems. Before generating the morph set, users may select ligands with a desired force field and choose the method to generate the perturbation network. For Amber-TI, GROMACS-NETI, and OpenMM-FEP, various types of perturbation networks are supported, including minimum spanning tree (MST), LOMAP,^36^ radial, and custom networks. Once a perturbation network is selected, the corresponding set of morph pairs can be generated automatically (Figure 2A). The resulting morphs can be viewed and modified in both tabular and graphical formats. In either case, users can find the corresponding core scaffold by hovering over the desired morph. A pairwise similarity matrix of ligands is also provided, allowing users to refine or update the generated network as needed. Once the set of morphs is determined, users can inspect and modify the core scaffold by clicking the core atoms in the 2D visualizer (Figure 2B, highlighted in red). In this study, we used custom networks for the JACS benchmark set, and BMS-776532/BMS791826 analogues (GR, 3BQD) and TNI-97 series (HDAC6, 5EDU) for the other two case studies. The MCS and LOMAP network generation involves identifying the MCS for every ligand pair, scoring each MCS using the LOMAP algorithm,^36^ and assembling the perturbation network according to the selected strategy. Assigned morphs are realigned based on their pairwise MCS, which also defines the core scaffold of the hybrid topology later on. For Amber-TI, the MCS is further refined to exclude any heavy atom bonded to a hydrogen atom to account for SHAKE constraints. Hybrid topologies for Amber-TI, GROMACS-NETI, and OpenMM-FEP are then constructed, followed by solvation, neutralization, and removal of overlapping water molecules of both the ligand-only and protein–ligand complex systems.

**Figure 2:**
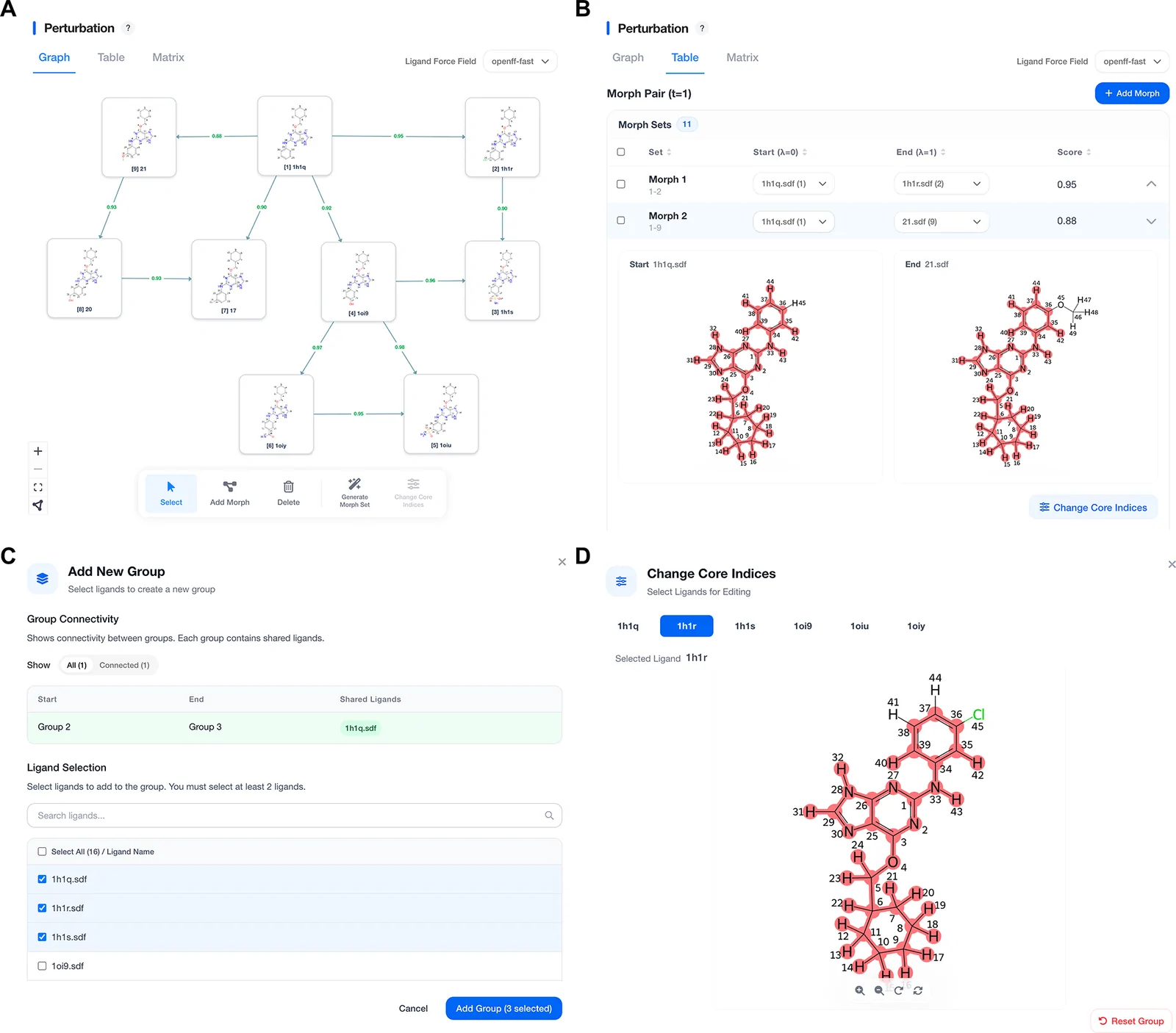
User interface of *MolCube-AFES*. Standard AFES calculation features of (A) automatically generated perturbation networks and (B) modification of core scaffold for each set of morphs. BLaDE-MSLD features of (C) selection of bridge ligand between groups and (D) modification of group-wise MCS.

For BLaDE-MSLD, *MolCube-AFES* supports a ligand grouping option, which proceeds as follows: (1) the MCS results of all ligands are computed to determine common cores across all ligands, (2) site-specific MCS results on substituents from (1) are calculated for every ligand pair, (3) ligands are grouped to maximize the common core with a user-defined maximum number of substituents per site, which can range from 2 to 9 (with a recommended value of 6). The resulting groups are *bridged* by sharing one common ligand to enable intergroup comparison of ΔΔ*G*_bind_. In this study, we expanded the maximum number of substituents per site to 20 and evaluated the performance of various combinations of ligand groups, sites, and substituents. Additional factors affecting simulation efficiency, such as minimal recommended changes per group and substituent volume, among others, are also considered empirically. Figure 2C,D illustrates the *MolCube-AFES* user interface displaying both pregrouped and grouped ligands for BLaDE-MSLD. The resulting ligand groups are then presented along with information, including the size of the core scaffold, the number of sites, and the number of substituents per site. Users may add, delete, or modify groups, ligands, or core atoms, as long as the groups maintain the *bridge* (Figure 2C). Hybrid ligand topologies are generated for each group by obtaining a group-wise MCS, realigning ligands accordingly, refining the MCS to remove atoms with differing bonded or nonbonded parameters, and performing charge renormalization (Figure 2D).^37^ The resulting topologies are then used to construct the ligand-only and protein–ligand complex systems, followed by solvation, neutralization, and removal of overlapping water molecules.

*MolCube-AFES* was not intended to replace the native backend workflows or method-specific setup protocols of Amber-TI, GROMACS-NETI, OpenMM-FEP, and BLaDE-MSLD. Rather, it provides a unified interface for preparing and executing these four free energy simulation methods under consistent system-setup conditions. Protein and ligand systems were processed using a common preparation module and force-field assignment procedure, enabling systematic cross-engine comparison while reducing differences introduced during initial structure and parameter preparation. This framework also lowers the practical barrier to applying specialized methods such as BLaDE-MSLD. *MolCube-AFES* performs alchemical system preparation through an independent topology and coordinate generation core, without relying on native external command-line tools such as OpenFE or pmx. For each ligand pair, the MCS is identified, atom mapping is performed, and a unified hybrid ligand template is constructed to include both mapped common atoms and end point-specific atoms. This representation supports structurally nontrivial transformations, such as ring-size changes in the PTP1B ligand set, by explicitly defining the common region and the atoms that appear or disappear in each end point state. The hybrid template is subsequently translated into the distinct input representations required by each supported backend, allowing the same ligand transformation to be evaluated across multiple free energy implementations with reduced variability from ligand mapping, topology generation, and system setup. Details of the preparation and simulation protocol for Amber-TI, GROMACS-NETI, OpenMM-FEP, and BLaDE-MSLD are provided in the subsequent subsections.

### 2.2 Thermodynamic Integration with Amber

TI is a widely used equilibrium method for calculating free energy differences by integrating the ensemble average of the derivative of the system Hamiltonian (*U*) with respect to an alchemical thermodynamic coupling parameter λ. The parameter λ ranges from 0 (initial state) to 1 (final state), interpolating between two chemical species. The free energy change is computed via:

$$\Delta G=\int_{0}^{1}{\left\langle \frac{\partial U\left(\lambda \right)}{\partial \lambda }\right\rangle }_{\lambda }\;d\lambda$$

ΔΔ*G*_bind_ between two ligands (L0 to L1) is calculated as

$$\Delta \Delta G_{\mathrm{bind}}^{L0\to L1}=\Delta G_{\mathrm{complex}}^{L0\to L1}-\Delta G_{\mathrm{ligand}}^{L0\to L1}$$

where Δ*G*_complex_^L0→L1^ and Δ*G*_ligand_^L0→L1^ represent the free energy changes associated with the alchemical transformations of ligand L0 to L1 in the protein–ligand complex and in ligand-only solution, respectively.

To accurately perform TI calculations, particularly for systems undergoing significant chemical transformations, the underlying representation of the molecular states is critical. Amber employs a hybrid topology scheme in which both the initial and final ligand states are presented simultaneously within the system with shared coordinates for common scaffold atoms, and their interactions with the environment are modulated by λ. By utilizing this framework, the system maintains separate, noninteracting topologies for each state, ensuring structural and energetic consistency throughout the transformation. As a result, it is particularly effective in handling complex chemical changes such as mutations or ligand modifications, while enabling accurate estimation of relative free energies by minimizing artifacts from topological discontinuities.

All Amber-TI molecular dynamics (MD) simulations were performed under periodic boundary conditions (PBCs) in the isothermal–isobaric (NPT) ensemble at 300 K and 1 atm. Temperature was maintained using Langevin dynamics with a collision frequency of 5 ps^–1^, while pressure was regulated with a Monte Carlo barostat.^38^ To enable the use of a 4 fs integration time step, covalent bonds involving hydrogen atoms were constrained using SHAKE algorithm, and hydrogen mass repartitioning (HMR) was applied.^39^ Nonbonded interactions were truncated at 9 Å. While advanced features such as smoothstep softcore^40^ and ACES-HREMD^41,42^ are available in Amber24, this study employed the standard softcore method used in Amber20, with the application of these advanced features further addressed in the CONCLUSIONS section. For simulations involving alchemical transformations, atoms undergoing modification were treated using a softcore potential,^15^ and van der Waals and electrostatic interactions in these regions were smoothly scaled during the transformation.

Prior to dynamics, all systems were subjected to energy minimization to remove unfavorable contacts. Minimization was carried out using a steepest descent algorithm for up to 1,000 steps. The softcore potential was applied during this stage to the transforming regions of the alchemical perturbation, and bond constraints were selectively removed from these atoms. Following minimization, the systems were gradually heated from 1 to 300 K over a 5 ps period using a linear temperature ramp. Heating was performed under NPT conditions using Langevin dynamics and the Monte Carlo barostat. This step ensured proper equilibration of the system prior to production. During heating, atoms involved in the alchemical transformation were excluded from bond constraints to allow for flexible structural adjustment.

Production simulations for Amber-TI were carried out using the Amber’s hybrid topology approach. Atoms present only in the initial or final ligand state were explicitly defined, and the softcore potential was applied throughout the transformation to avoid singularities. Each alchemical transformation was simulated across 12 discrete λ values (0.0000, 0.0479, 0.1151, 0.2063, 0.3161, 0.4374, 0.5626, 0.6839, 0.7937, 0.8850, 0.9521, and 1.0000).^43^ For each λ window, a 5 ns MD simulation was performed. To assess statistical variability, four independent replicates were conducted per transformation, and the average ΔΔ*G*_bind_ value is reported.^44^ During each simulation, energy evaluations for all λ states were recorded to facilitate multistate free energy estimation using both TI and the multistate Bennett acceptance ratio (MBAR) method.^45^ For subsequent comparisons with other methods, however, the MBAR-derived results were exclusively utilized.

### 2.3 Nonequilibrium Thermodynamic Integration with GROMACS

The GROMACS-NETI method leverages nonequilibrium dynamics and Jarzynski’s equality to estimate free energy differences from repeated fast switching simulations between initial and final states. The core idea is to generate multiple forward and reverse transformations, from which the free energy difference is extracted via exponential averaging:

$$\Delta G=-k_{B}T\;\ln\left\langle e^{-W/k_{B}T}\right\rangle$$

where *k*_B_ is the Boltzmann constant, *T* is temperature, *W* is the work done on the system during the nonequilibrium transformation, and the average is taken over many trajectories. This method avoids the need to sample intermediate λ-states, potentially reducing a simulation cost.

To enable these transformations between states, GROMACS employs a hybrid topology approach, which provides a consistent molecular representation throughout the simulation. In this framework, the scaffold atoms common to both the initial (L0) and final (L1) molecules are shared, while perturbed atoms are treated as “dummy” atoms that are gradually turned on or off using a coupling parameter λ. This λ parameter modulates the interactions (e.g., charges, van der Waals, bonded terms) of the atoms across a series of virtual states, allowing a smooth transformation between L0 and L1. The GROMACS hybrid topology method ensures a continuous transformation pathway and avoids complications from abrupt changes in atomic composition.

All NETI simulations were carried out using GROMACS 2025.2 with the PBCs applied. All simulations were performed in the NPT ensemble at 300 K and 1 bar. Temperature was regulated using the velocity-rescaling thermostat with a stochastic term (v-rescale), and pressure was maintained using the stochastic cell rescaling barostat (c-rescale). A 4 fs time step together with HMR was used, with all covalent bonds involving hydrogen atoms constrained to enable stable integration. Prior to alchemical simulations, all systems were subjected to energy minimization using the steepest descent algorithm to remove steric clashes and relax high-energy bad contacts. Following minimization, a two-stage equilibration protocol was applied. The system was first equilibrated under constant volume and temperature (NVT) conditions and then under NPT, with each phase conducted for 500 ps and 10 ns, respectively. During NVT equilibration, positional restraints were applied to heavy atoms to stabilize the system and preserve the overall structure.

Nonequilibrium alchemical transformations were performed with simulations initiated from equilibrated configurations at the two end states (λ = 0 and λ = 1). Alchemical regions were defined explicitly, and softcore potentials were applied to avoid singularities during the transition. Each transformation was executed using a linear pull of the λ-parameter over 250 ps, with forward and reverse runs of 100 replicates to ensure statistical convergence. Four independent trials were performed for each direction to improve statistical reliability. Free energy differences were estimated from the work distributions obtained in forward and reverse transformations. Postprocessing analysis was conducted using the *pmx* v.4.0.3 toolkit,^11,12^ which analyzed the nonequilibrium work trajectories to compute the final free energy estimates using the Crooks Gaussian Intersection (CGI), BAR, and Jarzynski estimators. Again, for subsequent comparisons with other methods, the BAR-derived results were exclusively utilized.

### 2.4 Replica Exchange Free Energy Perturbation with OpenMM

Similar to TI, alchemical FEP is a statistical mechanical approach for computing free energy differences between two thermodynamic states by introducing a nonphysical alchemical pathway that continuously connects them. In the traditional FEP framework, relative binding free energies between two ligands, L0 and L1, were computed by transforming ligand L0 into ligand L1 in the bound state. The relative free energy difference is defined as

$${\Delta G}_{L0\to L1}=-k_{B}T\;\ln\frac{Z_{L1}}{Z_{L0}}$$

where *Z*_L0_ and *Z*_L1_ are the configurational partition functions corresponding to the fully interacting states of ligands L0 and L1, respectively. Direct evaluation of this expression is impractical due to limited phase-space overlap between the end states; therefore, an alchemical coupling parameter λ is introduced to define a continuous transformation between the two states. Along this alchemical pathway, the system Hamiltonian is defined as

$$U\left(x:\lambda \right)=\left(1-\lambda \right)U_{L0}\left(x\right)+\lambda U_{L1}\left(x\right)$$

where *U*_L0_(*x*) and *U*_L1_(*x*) denote the potential energy functions of the initial and final ligand states, respectively, and *x* represents the full set of atomic coordinates. The free energy difference is obtained by sampling a discrete set of intermediate alchemical states λ_i_ spanning the interval from λ = 0 to λ = 1. In this study, however, FEP is used as a conventional umbrella term for alchemical relative free energy workflows, rather than as a designation of a specific estimator. Consistent with this usage, workflows such as Schrödinger FEP+ and OpenFE are referred to as FEP methods, although they employ MBAR for free energy estimation. Accordingly, we use the term OpenMM-FEP in this study to denote our OpenMM-based alchemical relative free energy workflow.

Efficient sampling of intermediate λ-states is essential for accurate free energy estimation, but is often hindered by slow conformational relaxation and poor configurational overlap between neighboring states, particularly near the end points of the alchemical transformation. To address these challenges, Hamiltonian replica exchange can be employed. In this approach, multiple replicas corresponding to different λ-states are simulated in parallel, and periodic exchange attempts of configurations between neighboring replicas are performed. Exchanges are accepted according to the Metropolis criterion,

$$P_{\mathrm{acc}}=\min\left[1,e^{-\beta \left(U_{i}\left(x_{j}\right)+U_{j}\left(x_{i}\right)-U_{i}\left(x_{i}\right)-U_{j}\left(x_{j}\right)\right)}\right]$$

where *β =* 1/*k*_B_T, *U_i_* and *U_j_* denote the Hamiltonians associated with λ*_i_* and λ*_j_*, and *x*_i_ and *x*_j_ are the configurations of replicas *i* and *j*, respectively. This exchange scheme enables configurations to diffuse efficiently across the alchemical space, thereby improving overlap between adjacent λ-states and accelerating convergence.

All alchemical FE calculations were performed using OpenMM hybrid system constructed in which the initial and final ligand states were combined into a single alchemical representation. Interactions corresponding to the two ligand states were smoothly interpolated as a function of λ, ensuring a continuous transformation while maintaining a physically consistent description of the system throughout the simulation. OpenMM-FEP is a custom alchemical backend implemented using OpenMM, rather than a direct interface to the full OpenFE toolkit. This implementation was developed to incorporate the validated alchemical potential formulations and force-energy functions used in OpenFE, while maintaining compatibility with the *MolCube-AFES* system-preparation and execution framework. The full OpenFE pipeline was not directly adopted because its network construction, graph execution, and workflow organization are designed around the OpenFE toolkit architecture, whereas *MolCube-AFES* requires a unified multibackend workflow shared across 4 methods. In this implementation, the alchemical energy functions were embedded into the native OpenMM-FEP backend and used with MolCube-generated topology and coordinate inputs. This design allows the OpenMM-based FE calculations to follow the same underlying physical formulation as OpenFE while preserving the programmatic flexibility required for MolCube-specific system preparation, including custom ligand mapping, hybrid topology generation, and complex system handling. Accordingly, OpenMM-FEP was implemented as a MolCube-native OpenMM backend that uses OpenFE-informed alchemical formulations but performs system preparation, input generation, and simulation execution independently of the full OpenFE workflow.

Hamiltonian replica exchange simulations were carried out using the alchemical utilities provided by *openmmtools.alchemy*.^46^ A series of intermediate λ-states was defined between the fully interacting initial and final ligand states. At each λ, an independent replica was propagated using Langevin dynamics at 300 K with a time step of 2 fs. Exchange attempts between neighboring λ-replicas were attempted every 2.5 ps. Each replica was equilibrated for 1 ns prior to production sampling. Following equilibration, production simulations of 5 ns per λ-state were performed. Configurations collected from all replicas were used for free energy analysis.

Relative free energy differences were estimated using the MBAR method. MBAR analysis was performed using the implementation available in *openmmtools.alchemy*, yielding statistically optimal free energy estimates by combining information from all λ-states. Convergence was assessed by examining overlap of reduced potential energy distributions between adjacent λ-states.

### 2.5 Multi-Site Lambda Dynamics with BLaDE

BLaDE supports MSLD, a dynamic alchemical approach in which λ-parameters are treated as additional degrees of freedom that evolve alongside atomic positions during simulation. Unlike traditional free energy methods such as FEP or TI, which have fixed λ values and require separate simulations for each state, MSLD enables the system to dynamically explore multiple alchemical states within a single trajectory. This makes BLaDE particularly effective for evaluating large combinatorial libraries of ligands with multiple substitution sites.

BLaDE, unlike GROMACS, does not support assigning multiple parameter sets, such as atom type, mass, and charge, to a single atom. This limitation introduces the need for additional steps, including core scaffold refinement and charge renormalization, to ensure accurate representation of atomic interactions. An initial core scaffold could be identified based on atomic connectivity and element type. However, the mapped core atoms may not always share identical force field parameters. To address this issue, in this study, the core scaffold is refined by excluding atoms with different bonded or nonbonded parameters, ensuring consistency within the core. Following the core scaffold refinement, charge renormalization is performed to maintain consistent core atom charges across ligands. For each core atom, partial charges are first extracted from all ligands and averaged. These averaged values are then reassigned to the corresponding core atoms. To preserve the original net charge of each molecule, the charges of the site (noncore) atoms are adjusted accordingly, ensuring overall charge neutrality and force field compatibility.^37^

To ensure accurate sampling and efficient convergence, BLaDE employs a biasing potential^47^ and adaptive landscape flattening (ALF) scheme^48,49^ that promote thorough exploration of alchemical space. A biasing potential flattens the free energy landscape and reduces barriers between alchemical states, and an adaptive scheme continuously updates this bias to promote transitions into under-sampled regions of λ-space, preventing the system from becoming trapped in low-energy basins and enabling uniform exploration across the combinatorial ligand set. Together, these techniques enhance the efficiency of sampling across a high-dimensional combinatorial space of substituents and ensure that free energy differences can be estimated with minimal statistical uncertainty.

Free energy differences are computed based on the time-averaged populations of alchemical states sampled throughout the trajectory, using reweighting techniques to correct for bias and ensure statistically robust estimates. The relative free energy between two alchemical states L0 and L1 is calculated using

$${\Delta G}_{L0\to L1}=-k_{B}T\;\ln\left(\frac{P_{L1}^{\mathrm{unbiased}}}{P_{L0}^{\mathrm{unbiased}}}\right)$$

where *P*_L0_^unbiased^ and *P*_L1_^unbiased^ are the reweighted, time-averaged populations of states L0 and L1, respectively. These were obtained by correcting the raw (biased) state populations *P*_*i*_^biased^ using the biasing potential *V*_bias_(λ_*i*_) applied during the simulation:

$$P_{i}^{\mathrm{unbiased}}\propto P_{i}^{\mathrm{biased}}e^{\beta V_{\mathrm{bias}}\left(\lambda_{i}\right)}$$

This reweighting ensures that the free energy estimates reflect the true underlying thermodynamic distribution, independent of the enhanced sampling strategy used to accelerate convergence. Therefore, the resulting free energies are statistically robust and account for uneven sampling due to adaptive biasing.

All BLaDE-MSLD simulations were conducted using *ALF* package^48^ with BLaDE^8^ as the MD engine, except during the energy minimization and short equilibration phase. Energy minimization and short equilibration was conducted using OpenMM (version 8.2.0) under the PBCs in the NPT ensemble at 300 K and 1 bar. Temperature was regulated using Langevin dynamics with a friction coefficient of 1.0 ps^–1^ and a time step of 2 fs, while pressure was maintained via a Monte Carlo barostat. Long-range electrostatics interactions were treated using the Particle Mesh Ewald (PME) method^50^ with a 9 Å nonbonded cutoff. All bonds involving hydrogen atoms were constrained to their equilibrium lengths. The system was minimized for up to 5,000 steps, followed by a 500 ps equilibration run employing simulated annealing to ensure adequate structural relaxation prior to subsequent ALF-based sampling.

Following energy minimization and short equilibration using OpenMM, all systems underwent a hierarchical ALF-based simulation protocol. Steps 1–111 comprised the flattening phase, consisting of 100 cycles of 26 ps equilibrium and 78 ps production followed by 11 cycles of 0.25 ns equilibrium and 0.75 ns production to iteratively refine the bias parameters. Step 112 consisted of 5 ns equilibration simulations with five independent trials (steps 112a–e), while Step 113 represented the production phase, consisting of 40 ns simulations for the protein–ligand complex (two trials; steps 113a,b) and 20 ns simulations for the ligand alone (five trials; steps 113a–e). Each production run was followed by postprocessing analysis to estimate the free energy change upon chemical perturbation using a histogram-based estimator. Rather than fixing the λ cutoff a priori, we determined it from the sampled λ trajectories as the largest value at which every alchemical endstate retained at least 10 frames in each independent trial. A single cutoff, taken as the minimum over all substituent groups and over both the complex and ligand legs, was applied throughout each system.

The BLaDE-MSLD simulation set up for λ-dynamics followed the general protocol of *ALF*.^48^ All simulations were performed under the PBCs within a cubic box in the NPT ensemble at 300 K and 1 atm. Temperature was maintained using a Langevin thermostat with a friction coefficient of 0.1 ps^–1^, while pressure was controlled using a Monte Carlo barostat^38^ with a maximum volume fluctuation of 100 Å^3^. The Leapfrog Verlet integrator was used with a 2 fs integration time step. Electrostatic interactions were computed using PME^50^ with an interpolation order of 6, Ewald coefficient of 3.125 Å^–1^, and a grid spacing of 1.1 Å. Nonbonded interactions were truncated at 9 Å. Hydrogen-containing bond constraints were enforced using the SHAKE algorithm. A softcore potential was applied during the initial 14 steps and deactivated thereafter. The ALF biasing function employed an exponential constant (λ_nexp_) of 5.5. Production runs were conducted under the same conditions as bias optimization. Note that, unlike Amber-TI and GROMACS-NETI, BLaDE-MSLD simulations in this study, we could not use HMR or the OPC water model due to the limitation of BLaDE topology scheme and the challenges associated with incorporating virtual sites in the current BLaDE implementation.

All BLaDE-MSLD simulations were executed within a single *ALF* project per system, with the exceptions of p38 and PTP1B. Since *ALF* natively supports up to 10 substituents per site, we recomputed the implicit-constraint free energy term G_imp_ to accommodate up to 20 substituents, thereby allowing every benchmark system to be represented as a single multiligand topology project. Both p38 and PTP1B systems with the highest MUE were simulated using two (PTP1B) and three (p38) separate *ALF* projects to assess potential performance improvements.

### 2.6 Evaluation Metrics

To quantify agreement between predicted and reference ΔΔ*G*_bind_, we report both absolute error and correlation-based measures, computed as per-target (per-protein) statistics averaged over the eight protein systems in the JACS benchmark. Root-mean-square error (RMSE) and mean unsigned error (MUE; equivalent to mean absolute error, MAE) measure absolute deviation between predicted and reference ΔΔ*G*_bind_ values in kcal/mol. Lower RMSE/MUE indicates better absolute agreement with the reference. The coefficient of determination (R^2^) and Pearson’s correlation coefficient (r_p_) evaluate whether a method preserves the relative ordering of ΔΔ*G*_bind_ values across transformations. Two correlation coefficients reflect target-specific structure–activity relationship trends. Finally, Acc ± 1 and Acc ± 2 report the fraction of predictions whose absolute error relative to the reference falls within ±1 and ±2 kcal/mol, respectively. These accuracy-in-window metrics provide a practical assessment of model performance by quantifying the proportion of affinity changes predicted within ranges that is generally regarded as informative for decision-making in compound design. Here again, values are averaged on a per-protein basis to prevent any single target from disproportionately influencing the results. Together, these metrics allow us to distinguish between two desirable aspects of performance: low absolute error (RMSE, MUE, Acc ± 1, and Acc ± 2), which demonstrates how close the predicted values are to the experimental measurements in terms of their magnitude, and high correlation (*R* ^2^, *r*_p_), which indicates how consistently the method preserves the correct binding ranking of ligand modifications within a series.

### 2.7 Bootstrap Statistical Analysis

To assess the statistical uncertainty of the performance metrics and the significance of pairwise method differences, we applied two complementary bootstrap resampling procedures (2,000 iterations each). First, 95% confidence intervals for each AFES method’s MUE were estimated by simple nonparametric bootstrap resampling. In each iteration, 80 morphs (JACS benchmark set) were drawn with replacement from the full data set, and MUE was computed on the resample. The 2.5th and 97.5th percentiles of the resulting distribution were taken as the 95% confidence interval. Second, to test whether any two methods differ significantly in MUE, we used a paired bootstrap procedure. In each iteration, the same set of 80 morphs, drawn with replacement, was used to compute MUE for both methods simultaneously, and the difference in MUE (ΔMUE = MUE_A_ – MUE_B_) was recorded. Resampling morphs in pairs preserves the morph-level correlation between the methods, which ensures that the difficulty of individual transformation affects the comparison. The 95% CI of ΔMUE was taken from its 2.5th and 97.5th percentiles, and a one-sided *p*-value was computed as the fraction of bootstrap samples in which the observed direction of the difference was not reproduced (e.g., fraction of resamples where ΔMUE ≥ 0). No correction for multiple comparisons was applied across the six pairwise tests.

### 2.8 Ensemble Combination Strategies

To evaluate whether combining predictions from multiple AFES methods improves accuracy, we tested four ensemble strategies applied to the mean predictions (averaged across four replicas) from each method. Equal weight averaging computes the ensemble prediction as the unweighted mean of the four method predictions. Inverse-MUE weighting assigns each method a weight inversely proportional to its global MUE (*w*_*i*_ = (∑_*j*_MUE_*j*_)/MUE_*i*_), so that more accurate methods contribute more to the ensemble prediction. Weights were computed from the global MUE of each method over all 80 morphs. Oracle ensemble represents an idealized upper bound on per-protein selection; for each protein, the method with the lowest MUE on that protein is selected, and its predictions are used exclusively. This is not a deployable strategy (as it requires knowledge of the ground truth), but serves as a reference for the maximum benefit achievable by perfect protein-level method selection. Linear stacking with leave-one-protein-out cross-validation (LOPO-CV) fits a linear combination of the four method predictions using ordinary least-squares, with weights constrained to be non-negative and sum to one. The weights were estimated using LOPO-CV to avoid overfitting on the 80-morph data set. In each fold, all morphs from one protein were held out, weights were fit on the remaining seven proteins, and predictions for the held-out protein were generated using those weights. This procedure was repeated for each of the eight proteins, and the resulting held-out predictions were used to compute the ensemble MUE. LOPO-CV ensures that the weights used to predict each protein were never trained on data from that protein, providing a realistic estimate of generalization performance.

## 3 Results and Discussion

### 3.1 JACS Benchmark

#### 3.1.1 Accuracy of Ligand Binding Free Energy Predictions

The performance of four alchemical free energy approaches (Amber-TI, GROMACS-NETI, OpenMM-FEP, and BLaDE-MSLD) was evaluated on the JACS benchmark consisting of eight protein targets and ten ligand transformations per target (80 total transformations in Table S1). For each method, the predicted ΔΔ*G*_bind_ were compared against the corresponding experimental binding affinity. Performance was assessed using two complementary views, namely scatter plots, which visualize how closely the predicted values track the reference values (Figure 3), and tabulated statistical metrics, which summarize agreement as per-target (per-protein) statistics averaged over the eight protein targets (Table 1). Note that the prediction results are the average ones from 4 independent runs in this study (see the raw data in AFES_Supplementary2.xlsx).

**Figure 3:**
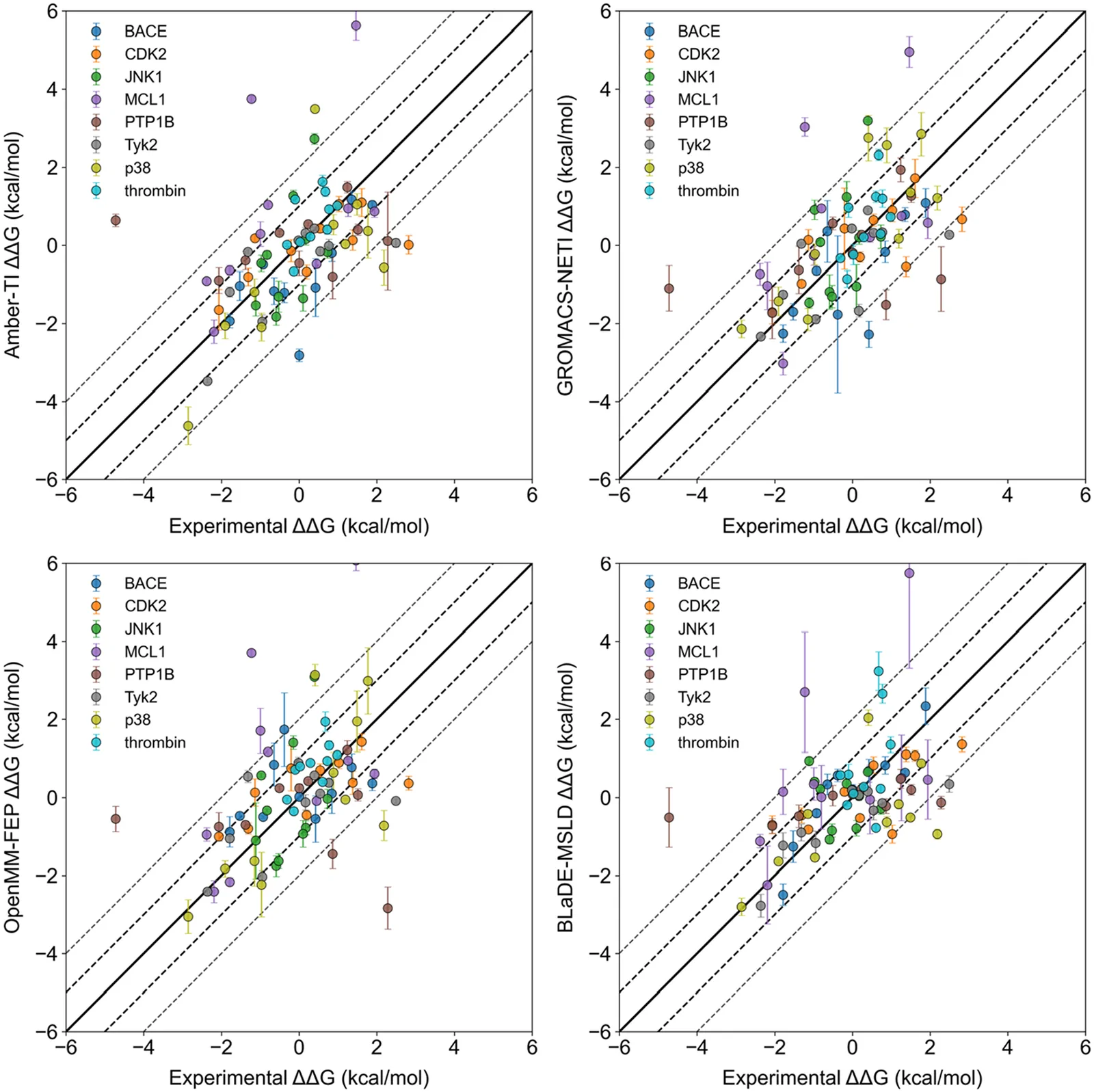
Comparison of ΔΔ*G*_bind_ prediction using *MolCube-AFES* (Amber-TI, GROMACS-NETI, OpenMM-FEP, BLaDE-MSLD) with the experiment results.

**Table 1:** Comparison of ΔΔ*G*_bind_ Values across the Four Methods with the Experimental Results

	RMSE (kcal/mol)	MUE (kcal/mol)	Correlation *R* ^2^	Kendall’s τ	Acc ± 1	Acc ± 2
Amber-TI	1.37	1.03	0.35	0.40	0.59	0.88
GROMACS-NETI	1.28	0.99	0.38	0.44	0.61	0.88
OpenMM-FEP	1.44	1.11	0.30	0.33	0.59	0.85
BLaDE-MSLD	1.24	0.96	0.43	0.38	0.66	0.90

The ΔΔ*G*_bind_ comparison scatter plots show that all four predictions cluster near the x = y line with 59–66% of transformations within ±1 kcal/mol and 85–90% within ±2 kcal/mol from experiment, indicating that all approaches capture both the direction and approximate magnitude of the binding affinity changes (Figure 3). Among the four methods, BLaDE-MSLD shows the lowest RMSE and MUE (1.24 and 0.96 kcal/mol; Acc ± 2 = 0.90), followed by GROMACS-NETI (1.28/0.99 kcal/mol; Acc ± 2 = 0.88), Amber-TI (1.37/1.03 kcal/mol; Acc ± 2 = 0.88), and OpenMM-FEP (1.44/1.11 kcal/mol; Acc ± 2 = 0.85).

All four methods produce extreme outliers particularly for MCL1 and PTP1B, as evident from the scatter plots, implying a higher risk of occasional large errors; also see Table S2. In the case of MCL1, the apparent outliers can be attributed to alchemical transformations that involve large structural perturbations, such as O → N atom substitutions within a fused 5.6-bicyclic ring or transformations of a benzene ring into a biaryl system (Amber-TI RMSE = 2.30 kcal/mol, MUE = 1.72 kcal/mol; GROMACS-NETI RMSE = 2.04 kcal/mol, MUE = 1.64 kcal/mol; OpenMM-FEP RMSE = 2.48 kcal/mol, MUE = 1.84 kcal/mol; BLaDE-MSLD RMSE = 2.11 kcal/mol, MUE = 1.64 kcal/mol). Similarly, for PTP1B, the observed outliers appear to arise from morphs that involve large structural perturbations, for example, when pronounced substituent rearrangements occur at a ring nitrogen, particularly in transformations introducing sulfur-containing, N-linked substituents, or when an additional seven-membered ring is introduced. Outliers are also associated with structurally small but electronically significant transformations, e.g., ring-bound H → Cl substitutions (Amber-TI RMSE = 2.02 kcal/mol, MUE = 1.43 kcal/mol; GROMACS-NETI RMSE = 1.76 kcal/mol, MUE = 1.25 kcal/mol; OpenMM-FEP RMSE = 2.32 kcal/mol, MUE = 1.62 kcal/mol; BLaDE-MSLD RMSE = 1.73 kcal/mol, MUE = 1.27 kcal/mol).

The numbers of ligands, sites, and substitutions used in the BLaDE-MSLD simulations are summarized in Table S3. Among the BLaDE-MSLD results, PTP1B exhibited particularly poor accuracy in its single-project form (Table S4 1 group MUE = 12.17 kcal/mol). Inspection indicated that the perturbations among its substituents were excessively heterogeneous, ranging from minimal fragments such as a methyl group to bulky bicyclic groups bridged by a sulfonamide, resulting in transformations that were not physically traversable within a single alchemical simulation (Figure 4). To address this, we performed multigroup simulations on the two worst-performing systems, p38 and PTP1B (Figure 4 and Table S3). Table S4 reports the computed ΔΔ*G*_bind_ values for each morph in the p38 and PTP1B systems. For PTP1B, dividing the ligands into two groups according to their volumetric and topological disparity led to a dramatic improvement (MUE 12.17 → 1.27; *r*_p_ −0.11 → 0.67 in Tables S2, S4). For p38, partitioning the ligands into three groups based on substituent site resulted in a modest gain (MUE 1.54 → 1.21; *r*_p_ 0.23 → 0.60 in Tables S2, S4).

**Figure 4:**
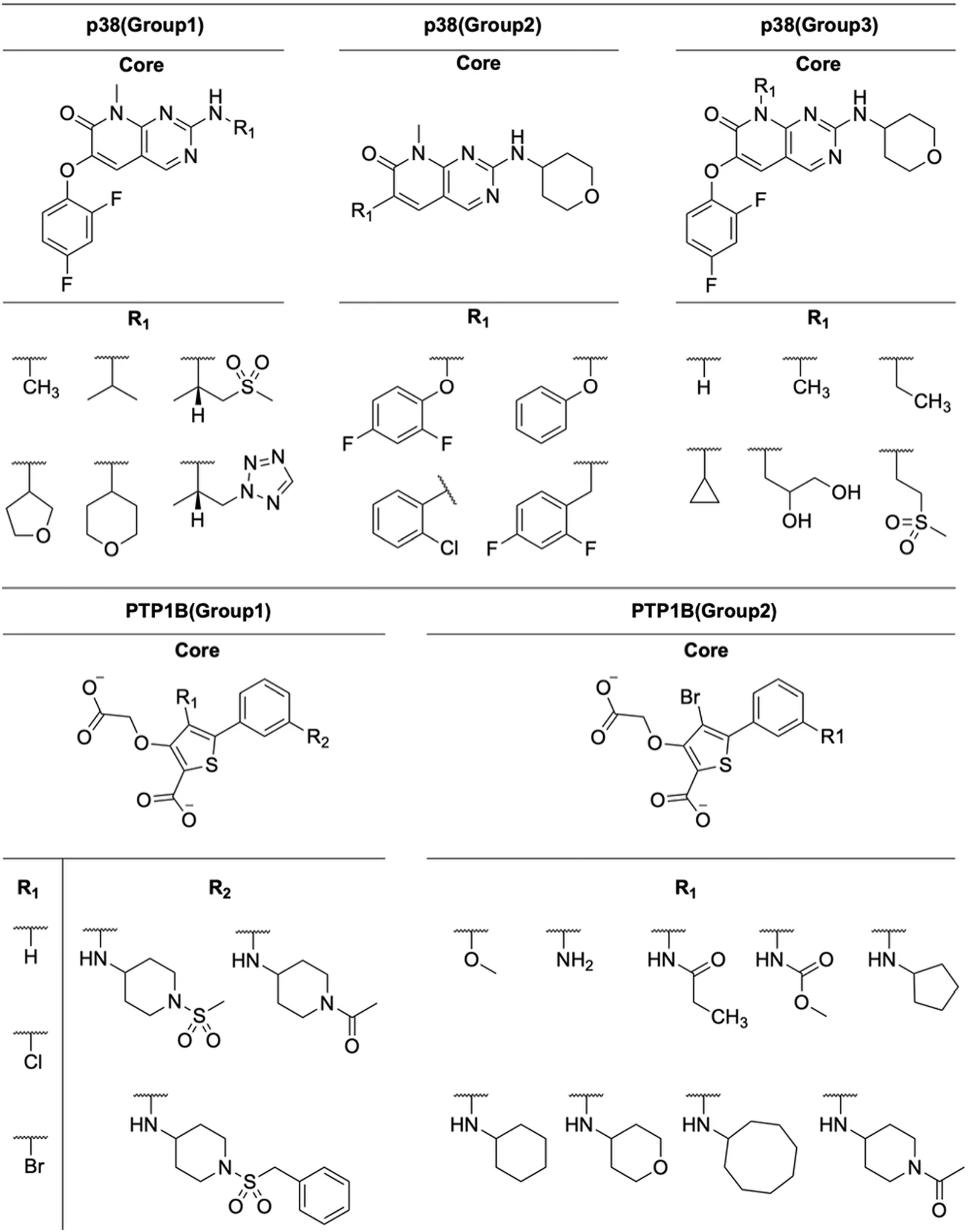
p38 (group 1, 2, and 3) and PTP1B (group 1 and 2) ligand structures with sites and substitution groups.

Among the four methods, BLaDE-MSLD, using the multigroup simulation strategy for p38 and PTP1B, achieves the best quantitative agreement with experiment across absolute-error metrics, attaining the lowest RMSE (1.24 kcal/mol) and MUE (0.96 kcal/mol) alongside the highest Acc ± 1 (0.66) and Acc ± 2 (0.90), and shows a relatively uniform distribution of errors with few extreme outliers.

All four methods exhibit statistically meaningful correlations with experiment, indicating that they capture the underlying structure–activity trends across the data set. OpenMM-FEP shows the lowest correlations (*R* ^2^ = 0.30, *r*_p_ = 0.48), with OpenMM-FEP exhibiting sensitivity to a few larger outliers. Amber-TI achieves moderate correlation (*R* ^2^ = 0.35, *r*_p_ = 0.56). BLaDE-MSLD and GROMACS-NETI attain the highest correlation coefficients (*R* ^2^ = 0.43, *r*_p_ = 0.57 and *R* ^2^ = 0.38, *r*_p_ = 0.61), reflecting more consistent preservation of rank ordering across ligand modifications. Taken together, BLaDE-MSLD provides the best overall combination of quantitative accuracy (lowest RMSE/MUE) and rank-ordering performance (highest *R* ^2^) across the JACS benchmark. The performance differences among the methods, however, are modest and their statistical significance is evaluated more rigorously in Section 3.1.4.

In addition to our internal evaluation, we also examined the agreement with the results reported by Wang et al. that used a widely adopted commercial workflow, *Schrödinger FEP+* (Figure S1 and Table S5). The RMSE and MUE reported by Wang et al. (1.23/0.99 kcal/mol) are comparable to those of GROMACS-NETI and BLaDE-MSLD (1.28/0.99 kcal/mol and 1.24/0.96 kcal/mol). Their Acc ± 1 value (0.57) is similar to those from our four methods (0.59–0.66), whereas Acc ± 2 (0.89) is nearly identical to that of GROMACS-NETI, Amber-TI, and BLaDE-MSLD (0.88–0.90). In terms of correlation, *Wang et al*. achieved *R* ^2^ = 0.40 and *r*_p_ = 0.62, somewhat similar with those of all four methods in this study (*R* ^2^ = 0.30–0.43). Overall, the commercial *FEP+* workflow and the open-source methods evaluated here achieve broadly comparable accuracy on the JACS benchmark, with BLaDE-MSLD offering the closest performance to *FEP+* among our implementations.

#### 3.1.2 Convergence Analysis

For the Amber-TI protocol, we performed convergence analysis to ensure that results obtained with 12 λ windows and 5 ns per window reflect equilibrated sampling. The λ-window overlap matrix and the forward and reverse ΔΔ*G*_bind_ for the 6e → 6b transformation of thrombin are shown in Figure 5A. As reported by Klimovich et al.,^51^ agreement between forward and reverse estimates within their uncertainties indicates a convergence of ΔΔ*G*_bind_; in our case, the two estimates are statistically consistent under the 12-window protocol. Previous studies further suggest that the λ-window overlap matrix should be at least tridiagonal, with diagonal and near-diagonal elements with ≥0.03, in order to obtain trustworthy free energies.^51^ This expected minimum-overlap structure is observed in our calculations, supporting both numerical stability and physical reliability of the TI-based calculations.

**Figure 5:**
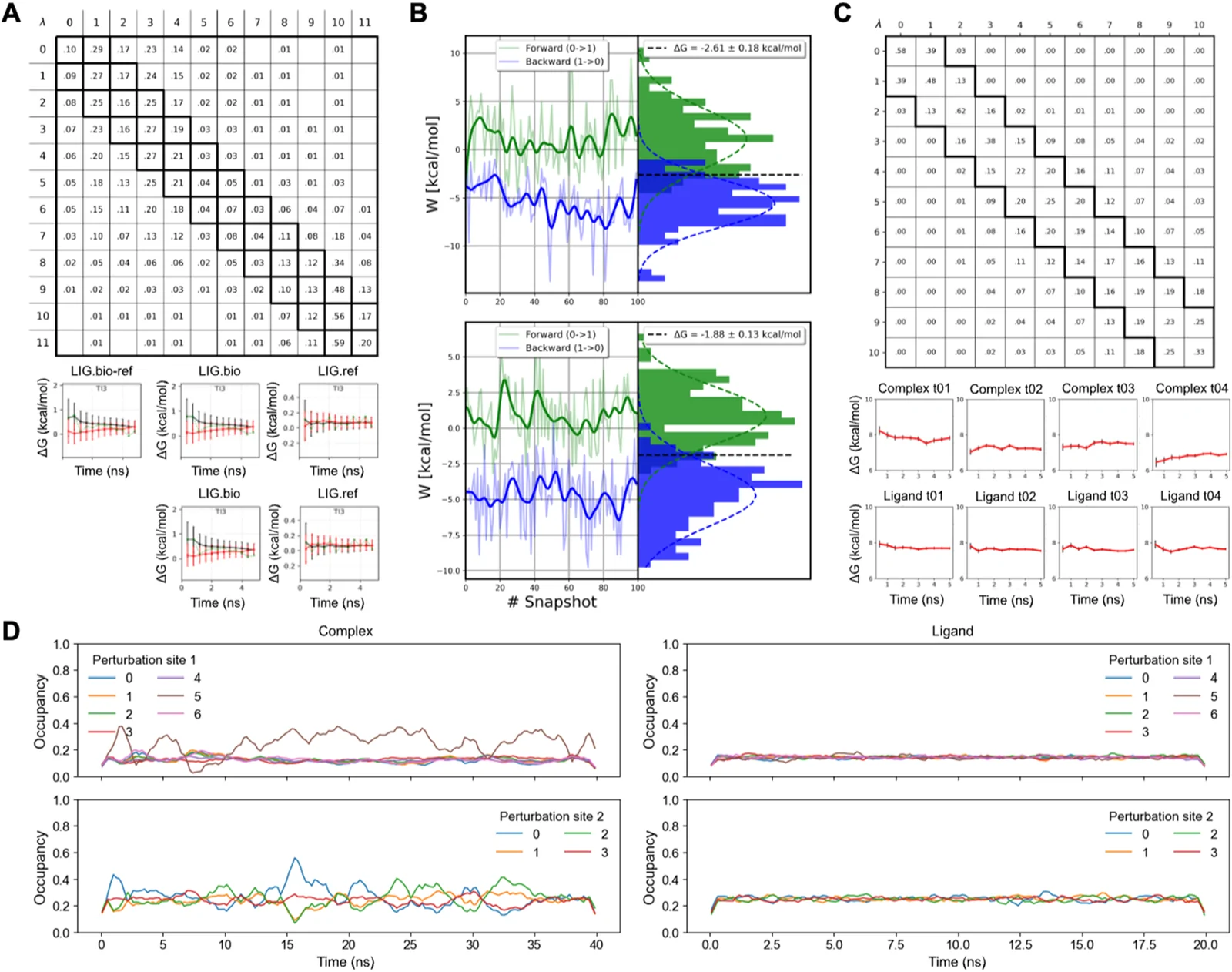
Sampling quality, convergence behavior, and state-space exploration evaluated using Amber-TI, GROMACS-NETI, OpenMM-FEP, and BLaDE-MSLD. (A) Amber-TI analysis results using a 12-window, 5 ns per window protocol: the λ-window overlap matrix and the time evolution of the forward (black solid line), reverse (red solid line), and block-averaged (green dotted line) estimates of Δ*G*_bind_ for each λ value. (B) GROMACS-NETI *pmx* analysis results: complex and ligand states corresponding forward (green) and reverse (blue) work distributions (bold lines for the mean work as a function of snapshot) together with the estimated free energy change and statistical uncertainty. (C) OpenMM-FEP replica exchange analysis using a 12-window, 5 ns per window protocol: overlap matrix using MBAR and the time evolution of Δ*G*_bind_ estimates for each λ value. (D) BLaDE-MSLD transition analysis: λ profiles at site 1 and site 2 of the thrombin system (complex and ligand) reflect ligand occupancy during the simulation.

In GROMACS-NETI, Δ*G*_bind_ between two alchemical states L0 and L1 is estimated separately for the complex and for the isolated ligand using nonequilibrium switching trajectories. For each transformation, the external work applied to drive the system from L0 → L1 (forward) or L1 → L0 (reverse) is integrated, yielding forward and reverse work ensembles, *W*_f_ and *W*_r_, from which *pmx* derives Δ*G* and its associated uncertainty. In Figure 5B, the forward (green) and backward (blue) nonequilibrium switching trajectories are shown together with bold lines indicating the mean work as a function of snapshot. The corresponding forward and backward work histogram are also presented, and the statistics of their overlap region is used to estimate Δ*G*, so that extent of this overlap provides a practical indicator of convergence. The two work distributions exhibit a substantial overlap and yield a well-defined crossing point, consistent with the Crooks fluctuation theorem,^52^ producing Δ*G* estimates with reported uncertainties on the order of ∼0.13–0.18 kcal/mol in these examples. The absence of heavy tails or strong skew further suggests that the switching is not excessively dissipative and that the trajectories are well behaved. Taken together, these diagnostics indicate that the NETI calculations for both the complex and the ligand are well converged and numerically robust under our simulation protocol.

For OpenMM-FEP, we analyzed the Hamiltonian replica-exchange simulations using *openmmtools*. To assess whether neighboring λ windows sample sufficiently similar configurations, we computed an MBAR-based per-λ overlap matrix (Figure 5C). Briefly, we collected configurations generated during replica exchange and extracted the corresponding reduced energies evaluated across all λ states. These multistate energy data were then combined using MBAR to quantify the extent to which configurations sampled at one λ can be reweighted to other λ states. The resulting overlap matrix provides a simple diagnostic of sampling continuity along λ, with large diagonal elements as expected, appreciable values between adjacent λ states indicating good window-to-window connectivity, and progressively smaller values for more distant λ pairs reflecting increasingly distinct ensembles. Overall, the pattern in Figure 5C supports adequate overlap between neighboring windows and suggests that the OpenMM-FEP free-energy estimates are not driven by gaps in sampling along λ.

BLaDE-MSLD employs a multisite λ-dynamics framework that enables simultaneous sampling of multiple substituents across predefined modification sites within a single simulation. Instead of performing pairwise alchemical transformations between ligand variants, substituent states are continuously modulated through λ-like order parameters that dynamically evolve along the trajectory. To characterize the sampling behavior, we monitored the time evolution of the effective occupancy of each substituent at individual sites (Figure 5D). Each trace represents the population (i.e., occupancy) of a given substituent state during the simulation. The distribution of occupancies provides direct insight into the thermodynamic and kinetic behavior of the system. When multiple substituents exhibit comparable occupancies, this indicates either small free-energy differences between states or continuous sampling across substituent space, reflecting efficient exploration of the available chemical configurations. In contrast, when a single substituent dominates the occupancy, this suggests a clear thermodynamic preference corresponding to a lower free-energy state. At the same time, the temporal fluctuations of the occupancies reveal the extent of dynamic sampling. Frequent changes in occupancy indicate that the system transitions between substituent states on the simulation time scale, whereas a lack of fluctuation would suggest kinetic trapping in a particular configuration. Therefore, analysis of substituent occupancies allows simultaneous assessment of thermodynamic weighting (through relative populations) and kinetic accessibility (through temporal fluctuations), providing a comprehensive picture of sampling behavior in BLaDE-MSLD simulations.

#### 3.1.3 Statistical Robustness of Performance Differences

With the well-converged ΔΔ*G*_bind_ from the four AFES methods, to assess the statistical reliability of the observed performance differences in depth, we applied a paired bootstrap resampling procedure (2,000 iterations) to compute 95% confidence intervals for MUE for each method, and to evaluate the significance of all pairwise MUE differences (Figure 6). The bootstrap confidence intervals for MUE span approximately 0.3 ∼ 0.4 kcal/mol for all four methods, reflecting the inherent variability in estimating accuracy from 80 transformations. BLaDE-MSLD yields the lowest point-estimate MUE (0.96 kcal/mol; [0.77, 1.17]) followed by GROMACS-NETI (0.99 kcal/mol; 95% CI [0.80, 1.20]), Amber-TI (1.04 kcal/mol; [0.81, 1.29]), and OpenMM-FEP (1.11 kcal/mol; [0.88, 1.36]). The confidence intervals for all four methods overlap substantially, indicating that the observed ranking should be interpreted with caution.

**Figure 6:**
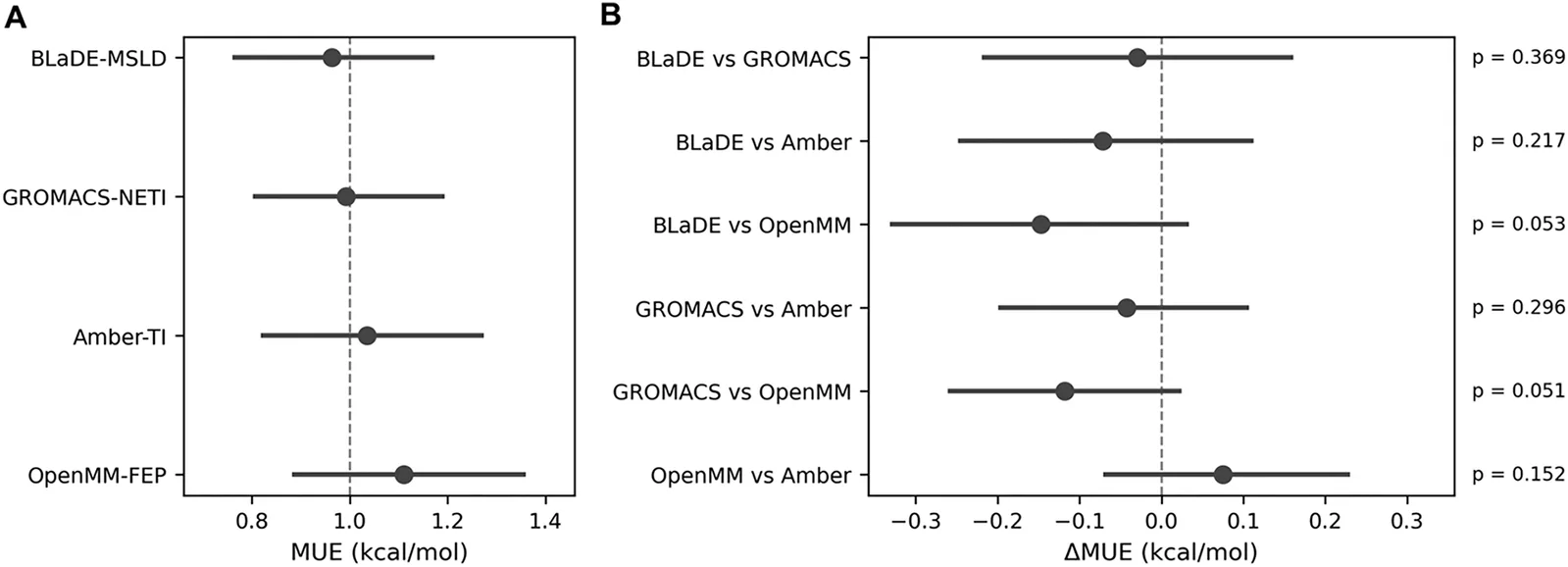
Bootstrap confidence intervals (95% CI, 2,000 iterations) for AFES method performance. (A) Point estimates and 95% CIs for MUE (kcal/mol) for each method. (B) Pairwise ΔMUE comparisons with 95% CIs and one-sided *p*-values. A vertical dashed line at zero indicates no difference.

Pairwise bootstrap comparisons confirm that no method differs significantly from any other at the conventional 0.05 level (one-sided *p*-values; Figure 6B). BLaDE-MSLD versus OpenMM-FEP has the most significant comparison (ΔMUE = −0.15 kcal/mol; 95% CI: [−0.25, + 0.03]), suggesting a modest trend favoring BLaDE-MSLD that is not yet strongly supported by the current data set size. All other pairwise differences are consistent with chance variation (*p* > 0.05). These results indicate that, while BLaDE-MSLD numerically outperforms the other approaches in the JACS benchmark, the four methods are statistically equivalent at the level of the present data set, and no definitive conclusion about superiority can be drawn from these 80 transformations alone.

#### 3.1.4 Effect of Combining Multiple Methods

Given the availability of predictions from four independent AFES approaches, we evaluated whether an ensemble of methods could provide more accurate predictions than any individual method alone (Figure 7). We compared three ensemble strategies; (i) equal-weight averaging of all four predictions, (ii) inverse-MUE-weighted averaging, and (iii) an idealized oracle that selects the best-performing method for each protein. As an additional reference, we also tested (iv) a linear stacking model trained via leave-one-protein-out cross-validation (LOPO-CV).

**Figure 7:**
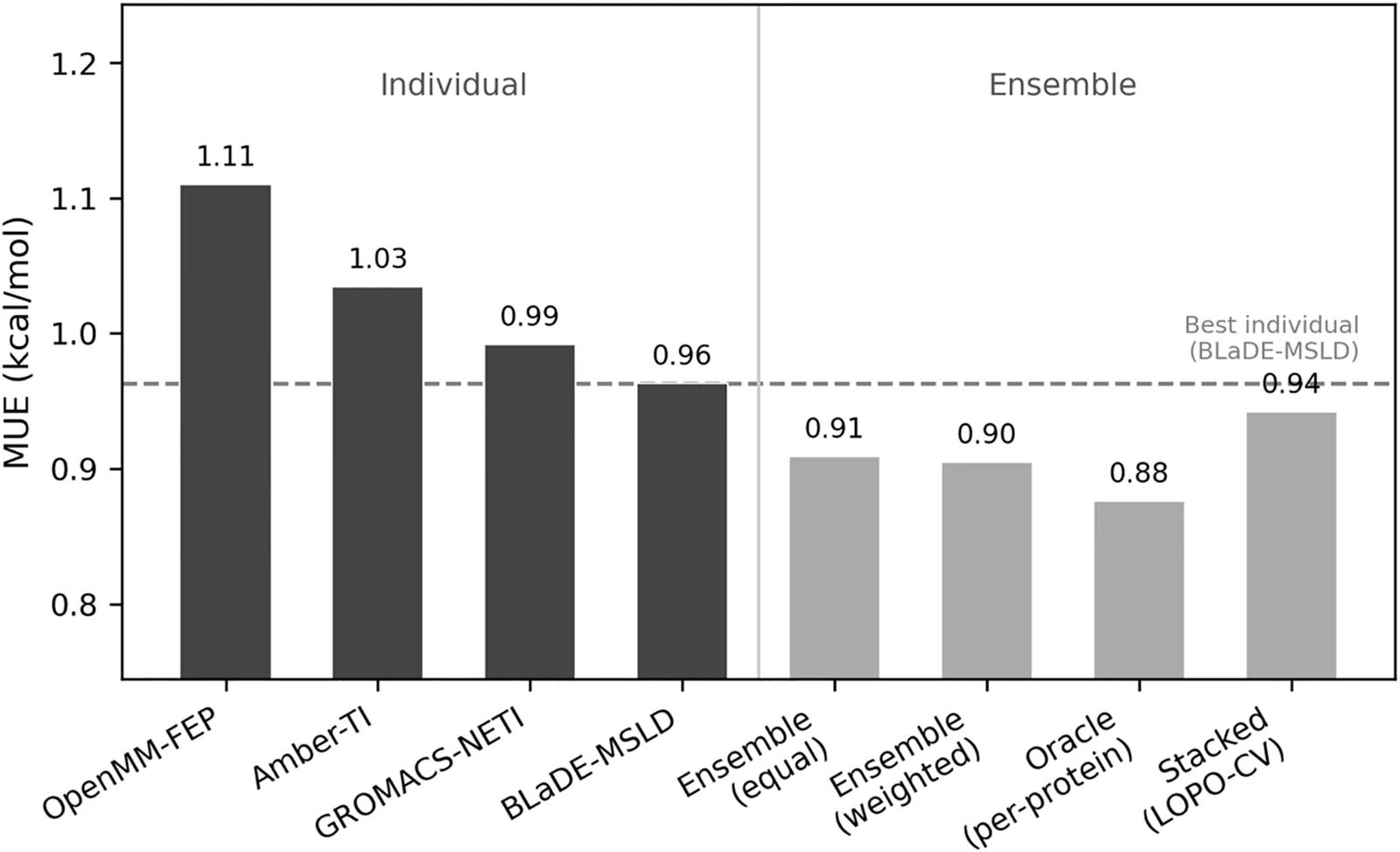
MUE for individual AFES methods and ensemble combination strategies. The dashed line indicates the MUE of the best individual method (BLaDE-MSLD, 0.96 kcal/mol). Ensemble strategies include equal-weight averaging, inverse-MUE-weighted averaging, an idealized oracle (best method per protein), and a linear stacking model (LOPO-CV).

Equal-weight ensemble averaging reduced MUE from 0.96 kcal/mol (best individual, BLaDE-MSLD) to 0.94 kcal/mol, a modest improvement of approximately 2%. Inverse-MUE weighting yielded a nearly identical result (0.90 kcal/mol), and even the oracle, which has access to protein-level ground truth for method selection, achieved only 0.88 kcal/mol, suggesting a ceiling on the benefit achievable by combining these four methods. The linear stacking model shows comparable MUE (0.94 kcal/mol).

The limited benefit of ensemble combination can be attributed to the strong intermethod correlation of the four predictions: Pearson correlation coefficient ranges from 0.66 (GROMACS-NETI vs BLaDE-MSLD) to 0.84 (OpenMM-FEP vs Amber-TI), indicating that the four methods largely agree on the same relative binding trends (data not shown). When individual predictions are highly correlated, averaging primarily reduces noise rather than systematic bias, yielding diminishing returns. In contrast, if methods with greater diversity were combined (e.g., those using different force fields or sampling protocols), the benefit of such an ensemble approach would likely be more pronounced. Exploring such heterogeneous ensembles represents an interesting direction for future work.

#### 3.1.5 Effect of Replica Averaging

Each of the four methods was run with four independent replicas. To evaluate the benefit of averaging multiple replicas, we systematically computed the mean MUE across all combinations of *k* replicas for *k* = 1, 2, 3, 4 (Figure 8). This analysis quantifies how much accuracy can be gained by running additional replicates per transformation.

**Figure 8:**
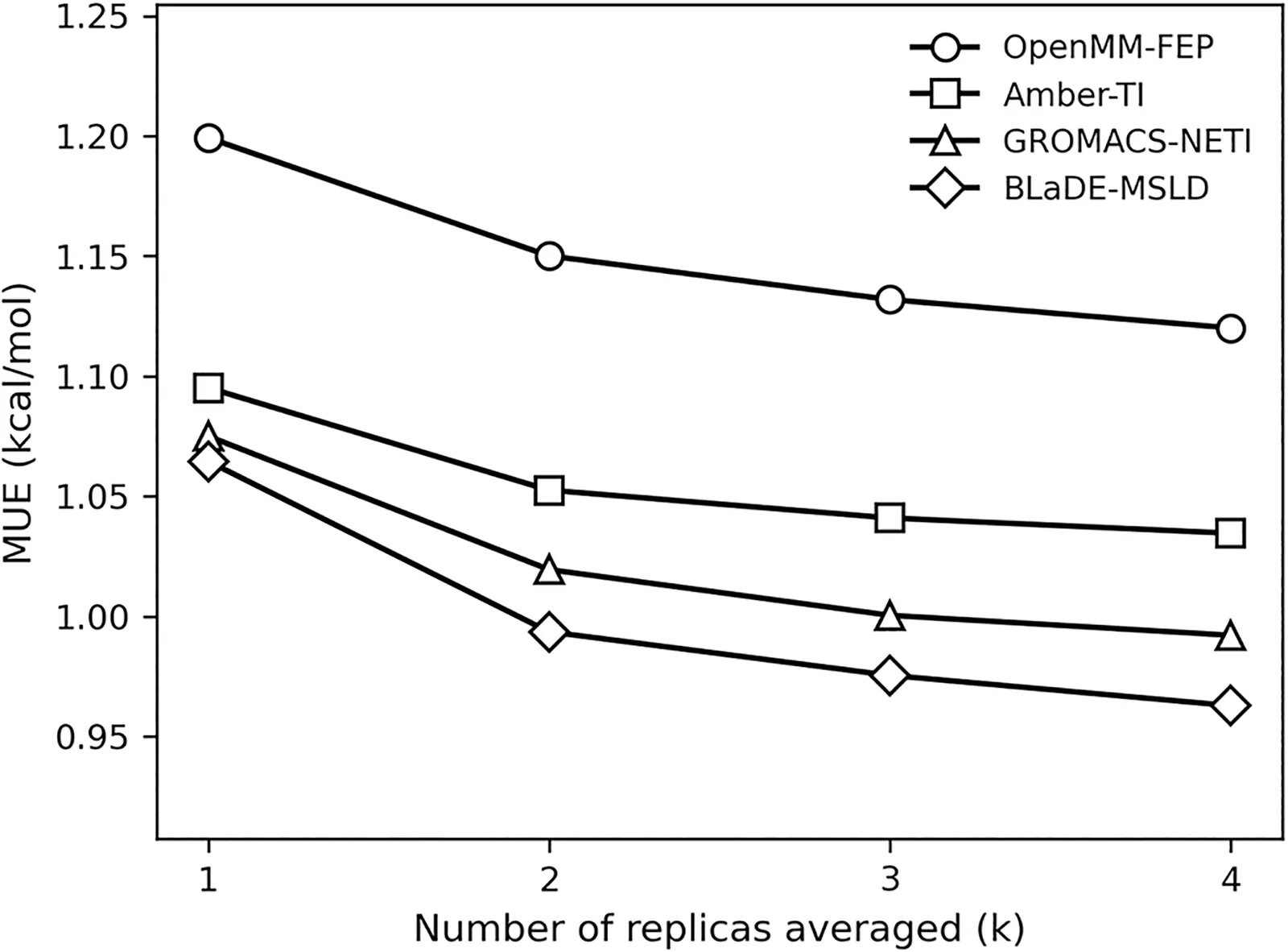
MUE as a function of the number of replicas averaged (*k* = 1, 2, 3, 4) for each of the four AFES methods, computed over all C­(4, *k*) replica combinations.

For all four methods, averaging replicas reduces MUE relative to a single replica, but the gains are modest. Going from one to four replicas improves MUE by 6 ∼ 11% across methods: BLaDE-MSLD shows the largest absolute gain (1.07 → 0.96 kcal/mol; 10.3%), followed by GROMACS-NETI (1.08 → 0.99 kcal/mol; 8.3%), OpenMM-FEP (1.19 → 1.11 kcal/mol; 6.7%), and Amber-TI (1.10 → 1.04 kcal/mol; 5.5%). The most substantial fraction of the total gain occurs in the first additional replica; the 1 → 2 step accounts for 50 ∼ 73% of the full 1 → 4 improvement across methods. In other words, beyond *k* = 2, the marginal benefit diminishes rapidly. These results suggest that a minimum of two replicas is advisible to capture the most impactful variance reduction, while the incremental benefit of running three or four replicas is relatively small given the additional computational cost.

#### 3.1.6 Computational Performance

To compare the computational performance of each AFES method, we first evaluated ten thrombin morphs (i.e., transformations) using Amber-TI, GROMACS-NETI, and OpenMM-FEP on an NVIDIA RTX A5000 GPU (Tables 2 and S6). The total wall-clock time is 135.51 h (13.55 h per morph) for Amber-TI, 118.34 h (11.83 h per morph) for GROMACS-NETI, and 122.08 h (12.21 h per morph) for OpenMM-FEP, indicating that the nonequilibrium protocol is approximately 9 ∼ 13% faster on average in our current simulation protocol. For every individual morph, the total runtime of GROMACS-NETI was shorter than that of Amber-TI and OpenMM-FEP, and the performance advantage was particularly pronounced for the ligand leg of the calculation (Table S6).

**Table 2:**
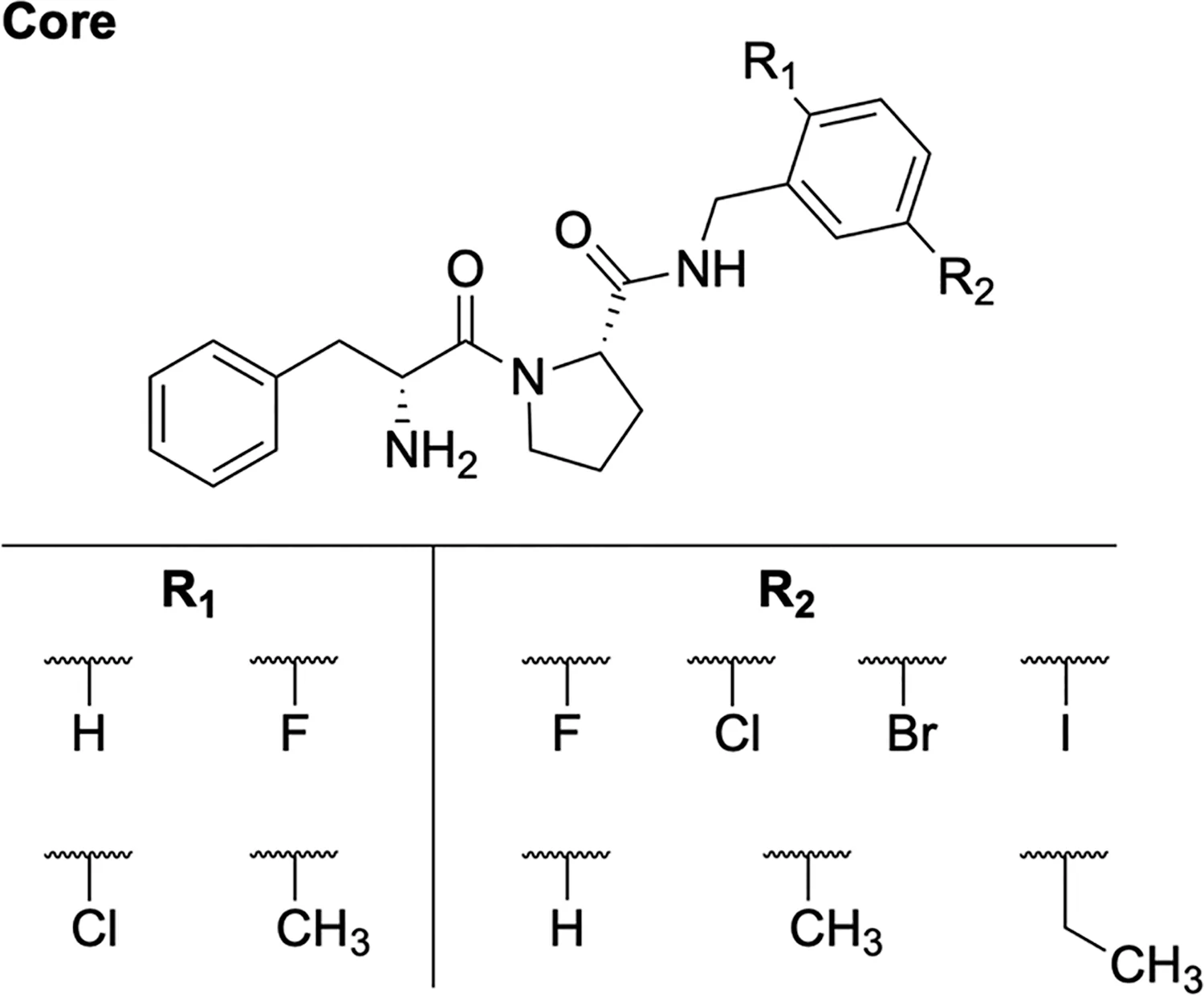
Performance Comparison of Four AFES Methods for Thrombin Using One NVIDIA RTX A5000 GPU

	Amber-TI (10 morphs)	GROMACS-NETI (10 morphs)	OpenMM-FEP (10 morphs)	BLaDE-MSLD (378 morphs with 28 substitution)
Total runtime (h)	135.51	118.34	122.08	26.80

In BLaDE-MSLD, a single multi-ligand simulation samples all 28 substitution combinations (4 from site R_1_ × 7 from site R_2_ in Table 2), and the relative free energies of the individual ligands are extracted from this trajectory. These per-ligand free energies can be combined to construct a total of 378 morph sets, corresponding to all possible unordered ligand pairs, without performing separate simulations for each pair. In other words, the 378 morphs are obtained by postprocessing a single simulation of all 28 substitution combinations from 10 ligands rather than 378 distinct alchemical transformations. The total wall-clock time required for this BLaDE-MSLD simulation is 26.80 h with 2 fs time step (n.b., GROMACS-NETI and Amber-TI used 4 fs time step with HMR in Table 2).

For the TI protocols and the FEP calculations, the mean total runtime averaged over 80 transformations in Amber-TI, GROMACS-NETI, and OpenMM-FEP is 12.53 h per morph. In contrast, BLaDE-MSLD characterized 378 morph sets in 26.80 h, which corresponds to an effective cost of approximately 0.07 h per morph. These results demonstrate that the MSLD approach provides substantially higher computational efficiency and throughput in terms of the number of ligand pairs that can be characterized per unit wall-clock time. However, this comparison reflects the observed throughput under the applied execution settings, and the effective turnaround time can change when independent simulations are allowed to run fully in parallel. Therefore, under the assumption of unrestricted GPU availability, the effective wall-clock turnaround time depends on the dependency structure and parallelizability of each AFES workflow. For Amber-TI, although the 12 λ windows can be distributed across available GPUs, the minimization, equilibration, and production stages within each λ window are sequentially dependent. Therefore, the effective turnaround time is determined by the longest per-window workflow and was estimated to be 1.13 h per morph. In GROMACS-NETI, the minimization and two equilibration stages are sequentially dependent on the preceding steps. However, the NETI calculations initiated from the 100 selected frames are mutually independent and can be executed fully in parallel when sufficient GPU resources are available, resulting in an estimated turnaround time of 0.72 h per morph. In contrast, OpenMM-FEP follows a sequential minimization, equilibration, and production workflow, and unlike Amber-TI, independent execution across λ windows was not applied in the current implementation. Thus, its effective turnaround time remains 12.21 h per morph, as derived from the total runtime in Table 2. Similarly, in BLaDE-MSLD, the minimization, flattening, equilibration, and production stages are all dependent on the outputs of the preceding stages, limiting parallel speedup at the workflow level. Consequently, under an idealized resource-unlimited scenario, GROMACS-NETI showed the shortest estimated turnaround time among the four AFES methods.

### 3.2 Case Study 1: Glucocorticoid Receptor

To demonstrate the practical application of *MolCube-AFES*, we conducted a case study using the glucocorticoid receptor (GR, PDB ID 3BQD), which was cocrystallized with deacylcortivazol.^53^ The 3BQD structure was originally used in the study by Weinstein et al. to model mode of azaxanthene-based selective GR modulators.^18^ This system serves as a benchmark due to the availability of a comprehensive experimental data set, comprising 13 compounds in total, including compound 14 as the reference, which cover a range of binding affinity (*K*_i_ values from 1.0 to 49.5 nM) reflecting modest energetic variations across various substituents.

In this study, we selected compound 14 from this series as the starting reference ligand because it possesses the most basic structure with an unsubstituted phenyl ring (R = H) (Table S7). However, because the protein–ligand complex structure with compound 14 is unavailable and the deacylcortivazol scaffold differs significantly from the azaxanthene core, identifying an appropriate binding mode is essential. Specifically, deacylcortivazol consists of five fused rings with a negative surface potential, whereas compound 14 features a three-ring core with a sulfur-containing thiazole and a more positive charge distribution. Due to these fundamental structural differences, we utilized Boltz-2 ^28^ to generate the binding pose for the receptor-compound 14 complex. Although Boltz-2 provides a useful protein–ligand complex model in the absence of an experimental cocrystal structure, AI-predicted ligand poses may still contain local geometric defects that are problematic for downstream free energy calculations.^54^ Since ΔΔ*G*_bind_ results can be sensitive to the initial ligand pose and geometry, we treated the Boltz-2 structure as an initial model rather than a directly finalized simulation input. The predicted complex was therefore passed through the LRM described in Section 2.1. In this step, atom overlaps are automatically detected and reported, and the protein–ligand complex is subjected to a short energy minimization to remove local strain and produce a physically reasonable starting structure. Minor overlaps are typically relieved during the standard minimization stage, while severe clashes or abnormal geometries are flagged for user inspection before generation free energy simulation inputs. This preprocessing step reduces the risk that artificial strain or local geometric artifacts in the Boltz-2 ligand pose propagate into the calculated ΔΔ*G*_bind_ values.

For this case, subsequent analysis of the generated structure revealed a distinct binding mode (Figure 9). While the interaction with GLN-570 was preserved, the polar interactions with ASN-564 and GLN-642 observed in 3BQD were lost as these residues encountered the hydrophobic regions of compound 14. Instead, new critical interaction was established with ARG-611. The predicted orientation indicates that while compound 14 occupies the same binding site as deacylcortivazol, its unique scaffold leads to a reorganized interaction network within the pocket. Therefore, its ligand geometry and binding mode consistency were examined and relaxed before being used for free energy setup.

**Figure 9:**
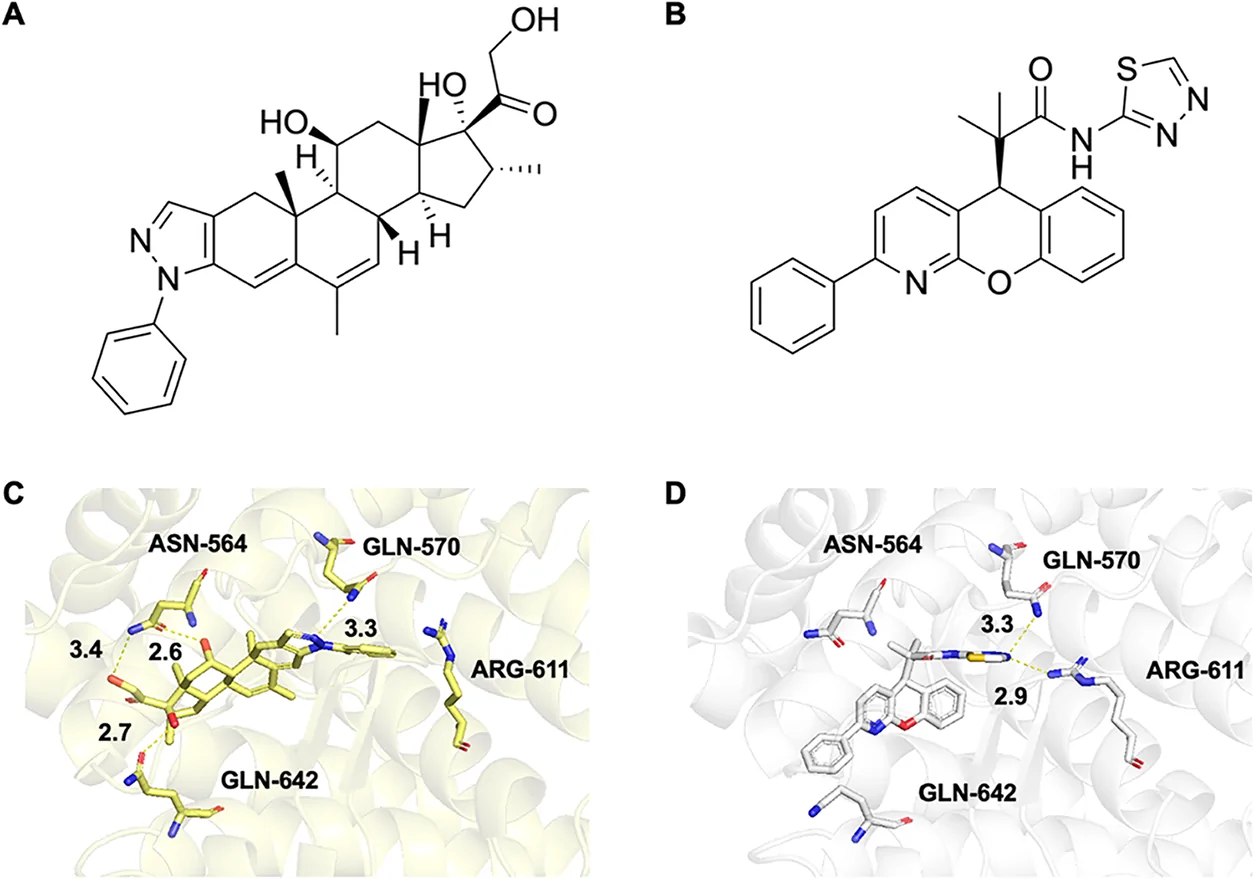
Structural comparison between the native ligand and the modeled compound. (A) 2D structure of deacylcortivazol, the cocrystallized ligand in PDB ID 3BQD. (B) 2D structure of compound 14, selected as the starting reference ligand for ΔΔ*G*_bind_ calculations. (C) Detailed binding mode of deacylcortivazol in 3BQD, showing key polar interaction with residues ASN-564 and GLN-642. (D) Predicted binding mode of compound 14 generated by Boltz-2. The interaction with GLN-570 is preserved, whereas contacts with ASN-564 and GLN-642 are lost and a new critical interaction with ARG-611 is established.

A total of 13 ligands with three substitution sites were tested, featuring various substitutions including alkyl chains (methyl, ethyl, propyl), heteroatom-containing groups (methoxy, isopropoxy, dimethylamino, hydroxy), and carbonyl derivatives (acetyl, isopropanoyl) (Table S7). To enhance the accuracy of simulation, the ligands were grouped based on their structural similarity by considering both the substitution sites and the chemical nature of the substituents to guide perturbation selection in the Amber-TI, GROMACS-NETI and OpenMM-FEP calculations. This classification reflects gradual changes in steric bulk and polarity, providing a practical framework for consistent comparison of calculated binding free energies while maintaining numerical stability in AFES. A comprehensive alchemical perturbation network was constructed connecting compounds 14 through 28 in multiple closed cycles (Figure S2). Crucially, each perturbation link (e.g., 14 to 15) was carefully designed to maximize structural overlap and enhance computational efficiency by representing the smallest feasible systematic substitution of functional groups (like Me or OMe), reflecting the gradual chemical variation within the congenic series. For the BLaDE-MSLD calculations, all ligands were handled as a single group, and the same set of perturbation networks in Figure S2 was used for the comparison with Amber-TI, GROMACS-NETI, and OpenMM-FEP calculations.

The predicted ΔΔ*G*_bind_ are compared with the experimental data for a congeneric ligand series in Figure 10. All four computational methods showed MUE values below 1 kcal/mol. Among them, BLaDE-MSLD shows the lowest absolute errors (RMSE = 0.90 kcal/mol, MUE = 0.63 kcal/mol), whereas OpenMM-FEP yielded results comparable to other standard AFES methods (RMSE = 1.07 kcal/mol, MUE = 0.98 kcal/mol). Although absolute error-based analysis shows apparent agreement with experiment, the coefficients of determination (*R* ^2^) are low for all methods and are not considered reliable indicators of model accuracy (Table 3). This behavior is primarily attributed to the modest experimental ΔΔ*G*_bind_ variation of the congeneric ligand series, with −2 to +2 kcal/mol. Given this limited energetic spread, low MUE values are expected and should be interpreted with caution, as they partly reflect the small dynamic range of the experimental data.^55,56^ Therefore, rather than relying on the low MUE or near-zero *R* ^2^ values as direct measures of predictive accuracy, this case study is most informative in showing that the four AFES methods using the Boltz-2 predicted ligand pose produced broadly comparable absolute-error performance within the experimental energetic scale of the data set.

**Figure 10:**
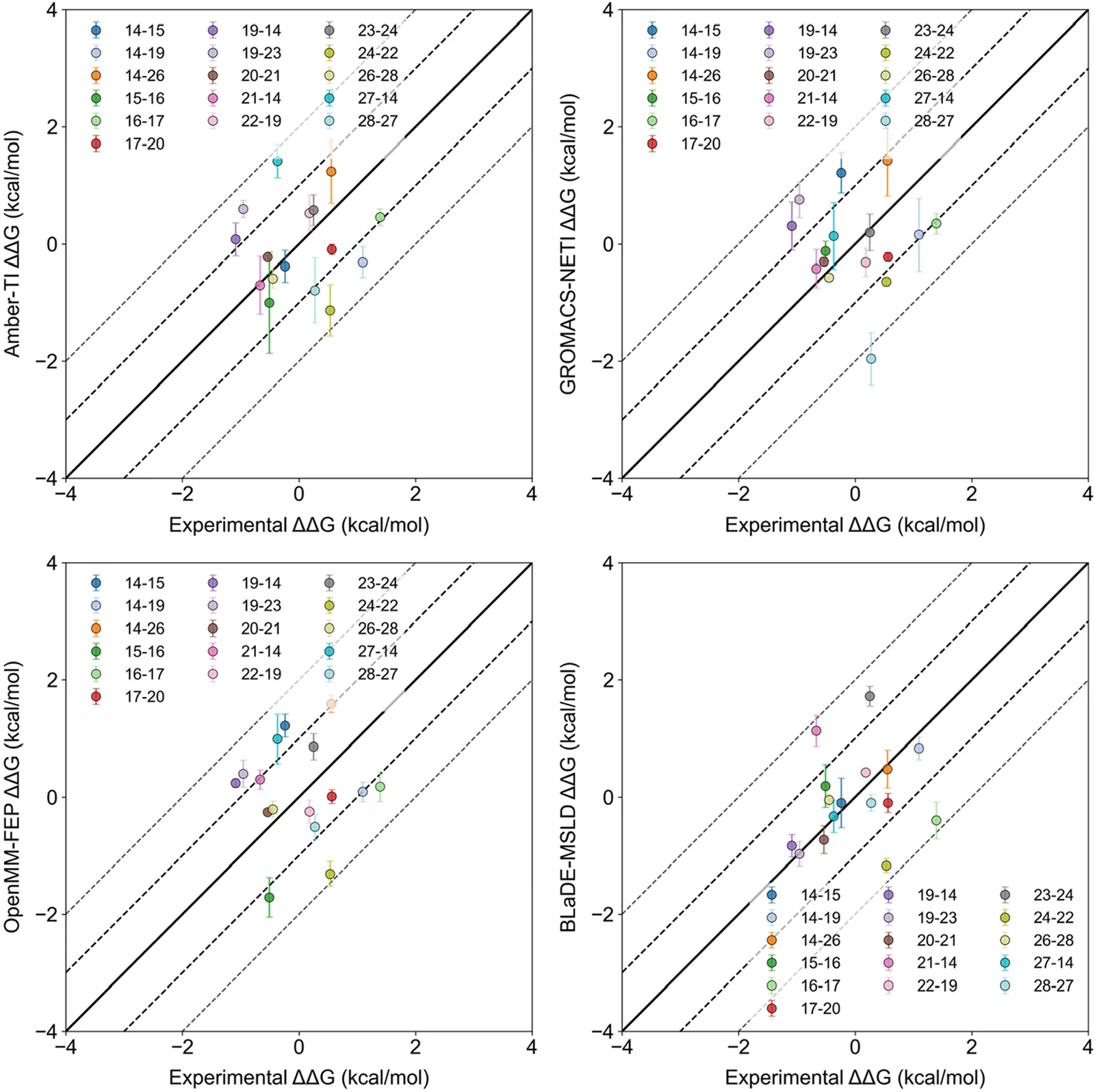
ΔΔ*G*_bind_ prediction-experiment for the glucocorticoid receptor case study.

**Table 3:** Comparison of MUE, RMSE, and *R* ^2^ for the Glucocorticoid Receptor Case Study

	RMSE (kcal/mol)	MUE (kcal/mol)	Correlation *R* ^2^
Amber-TI	0.98	0.79	0.01
GROMACS-NETI	1.04	0.85	0.00
OpenMM-FEP	1.07	0.98	0.00
BLaDE-MSLD	0.90	0.63	0.05

### 3.3 Case Study 2: HDAC6 Inhibitor

The study by Wang et al.^19^ reported TNI-97 as a highly selective and orally bioavailable HDAC6 inhibitor with promising activity against triple-negative breast cancer. HDAC6 was therefore selected as the target for our AFES benchmark, as the TNI-97 series (18 compounds: 30a-30r in Table S8) offers a well-defined and experimentally characterized data set with reported IC_50_ values suitable for rigorous computational evaluation. For the structural basis of the simulations, we employed the docking structure of HDAC6 with compound 30a (based on PDB ID 5EDU)^57^ provided by the original authors (Figure 11). All ligands in the series share a conserved 5-pyrazolyl-1H-benzotriazole scaffold appended with a terminal hydroxamate group responsible for zinc coordination, while systematic substitutions around the benzotriazole and phenyl moieties were introduced to modulate isoform (HDAC1) selectivity (Table S8). To ensure robust ΔΔ*G*_bind_ calculations, we constructed an alchemical perturbation network connecting compounds 30a through 30r in a closed cycle, enabling internal consistency (Figure S3).

**Figure 11:**
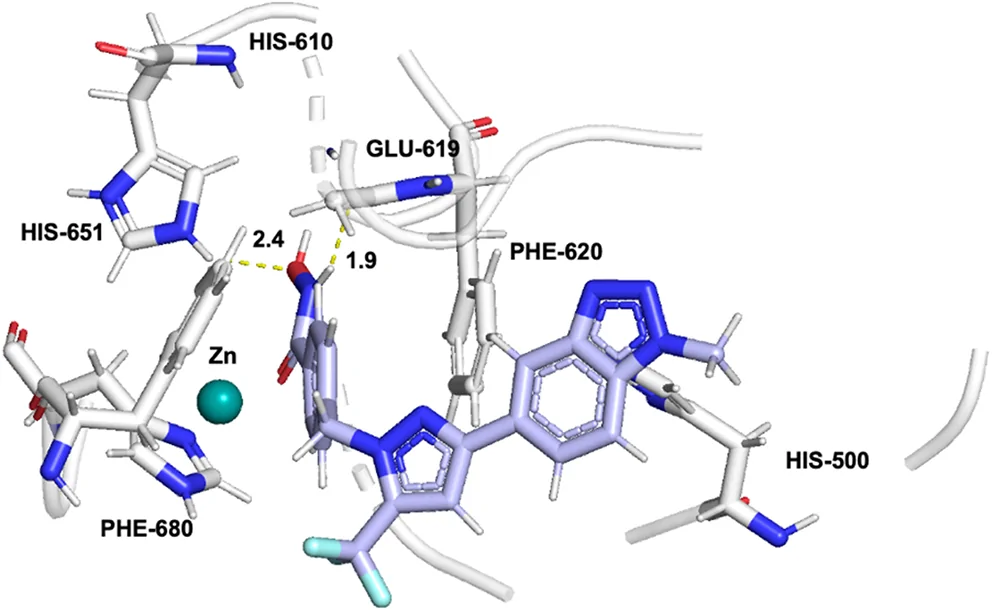
Docking structure of HDAC6 (based on PDB ID 5EDU) with compound 30a.

The predicted ΔΔ*G*_bind_ from all four methods exhibit MUE values in the range of 1.5–1.8 kcal/mol (Figure 12 and Table 4). While this level of deviation necessitates caution regarding absolute quantitative prediction, the MUE is commensurate with the typical uncertainty of binding assays. All four methods reproduced similar qualitative ranking trends for the subset of ligands, although global correlations were limited by specific outliers. Consequently, the global *R* ^2^ values are 0.08 or below for all methods, reflecting substantial deviations from experimental values in a subset of morphs involving a 30a ligand, rather than a systematic deficiency of the methods themselves (see the raw data in AFES_Supplementary2.xlsx).

**Figure 12:**
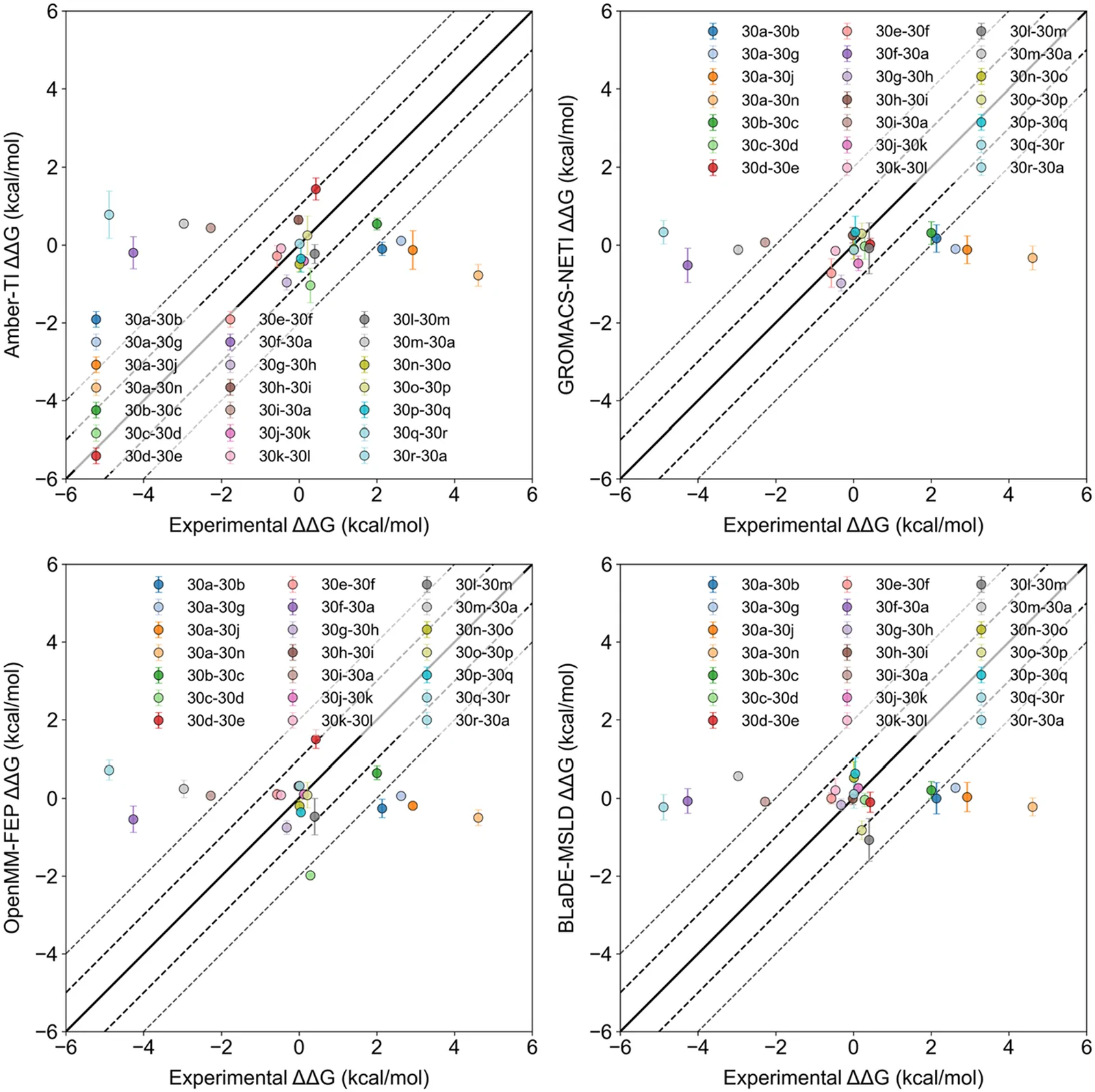
ΔΔ*G*_bind_ prediction-experiment for the HDAC6 inhibitor case study.

**Table 4:** Comparison of MUE, RMSE, and *R* ^2^ for the HDAC6 Inhibitor Case Study

	RMSE (kcal/mol)	MUE (kcal/mol)	Correlation *R* ^2^
Amber-TI	2.44	1.76	0.08
GROMACS-NETI	2.23	1.54	0.00
OpenMM-FEP	2.39	1.75	0.02
BLaDE-MSLD	2.25	1.65	0.00

Transformations involving 30a exhibit the most significant deviations from the experimental values. For instance, in the case of 30a to 30b transformation, the computed ΔΔ*G*_bind_ was nearly zero, a result physically consistent with the high solvent accessibility of the perturbation site. Despite this, the experimental data show a pronounced affinity difference (2.13 kcal/mol). This discrepancy suggests that the experimental trend may not arise from direct protein–ligand interactions but could instead be an artifact of binding mode instability or specific assay conditions unique to 30a. While simulations reached adequate convergence, the stark contrast with experimental data suggests that the latter may reflect complex structural transitions or solvent effects that challenge the standard interpretation of binding affinity at this position.^55,56^

To quantitatively isolate the source of this inconsistency, we converted the relative binding free energies (ΔΔ*G*_bind_) into the absolute binding free energies (Δ*G*_bind_) to allow direct comparison with experimental data across multiple simulations. All absolute free energies were reconstructed from the relative free energy network by solving an overdetermined system of linear equations using a least-squares approach with a zero-mean gauge constraint. In addition, a single global offset was applied for comparison with the experimental data. Consequently, converting ΔΔ*G*_bind_ to Δ*G*_bind_ led to a significant improvement in predictive accuracy, and 30a becomes more clearly identifiable as an outlier (see the raw data in AFES_Supplementary2.xlsx). Upon conversion to Δ*G*_bind_, the error metrics improved to RMSE/MUE values of 1.32/1.02 kcal/mol for GROMACS-NETI, followed by BLaDE-MSLD (RMSE = 1.36 kcal/mol, MUE = 1.17 kcal/mol), Amber-TI (RMSE = 1.51 kcal/mol, MUE = 1.20 kcal/mol), and OpenMM-FEP (RMSE = 1.56 kcal/mol, MUE = 1.33 kcal/mol) (Figure 13). These reduced error metrics indicate that the earlier discrepancies in ΔΔ*G*_bind_ were largely artifacts of error propagation from the 30a outlier rather than a systematic deficiency of the AFES methods themselves.

**Figure 13:**
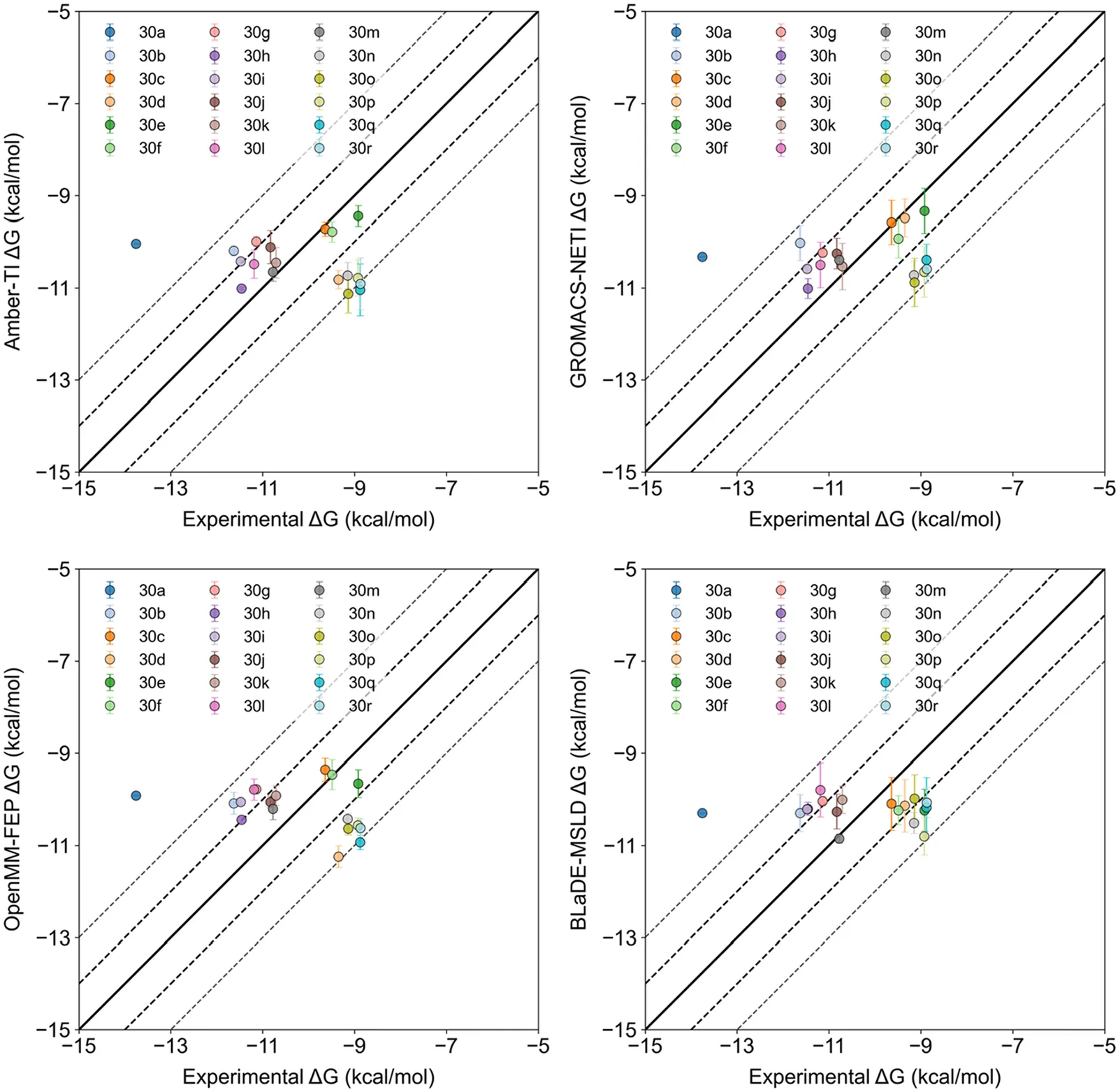
Absolute binding free energy (Δ*G*_bind_) prediction-experiment for the HDAC6 inhibitor case study.

## 4 Conclusions

The comparative evaluation of Amber-TI, GROMACS-NETI, OpenMM-FEP, and BLaDE-MSLD for the JACS benchmark set and 2 case studies (GR and HDAC6) has revealed broadly comparable performance profiles, with quantitative differences that are modest in magnitude. For the JACS benchmark set, BLaDE-MSLD achieved the lowest MUE (0.96 kcal/mol), closely followed by GROMACS-NETI (MUE = 0.99 kcal/mol), Amber-TI (MUE = 1.03 kcal/mol), and OpenMM-FEP (MUE = 1.11 kcal/mol). Importantly, bootstrap analysis (2,000 iterations) demonstrates that the 95% confidence intervals for all four methods overlap substantially, and pairwise differences are not statistically significant (*p* > 0.05 for all comparisons). These findings indicate that the four methods are statistically equivalent in the present benchmark and that observed ranking differences reflect sampling variability rather than systematic performance differences. Across all methods, predictions capture experimental trends within the margin of error that support actionable interpretation in a drug-discovery context.

Given the statistical equivalence of the four methods, practical method selection should be guided by workflow requirements rather than expected accuracy differences. GROMACS-NETI is preferable when robust rank ordering is the primary decision metric, attaining the highest Kendall’s τ (0.44), closely followed by Amber-TI (τ = 0.40). OpenMM-FEP offers broad compatibility through its open-source ecosystem and is appropriate when portability and extensibility are priorities. BLaDE-MSLD remains the method of choice for high throughput evaluation of extensive ligand sets because MSLD enables simultaneous sampling of many candidates in a single simulation, improving turnaround without sacrificing the quality required for screening. It also attains the lowest RMSE (1.24 kcal/mol), MUE (0.96 kcal/mol), and the highest *R* ^2^ (0.43) among the four methods, although its Kendall’s τ (0.38) is lower than that of GROAMCS-NETI and Amber-TI. Overall, method selection should align with the decision metric of interest, the anticipated magnitude of binding affinity changes, and the computational constraints of the project.

Ensemble averaging across the four methods yields only a modest improvement. About 2% MUE is reduced relative to the best individual method. This is consistent with the high intermethod correlation observed throughout the benchmark (Pearson *r* = 0.66–0.84). This limited gain implies that the four approaches share common systematic error sources (most likely force field parameters and perturbation network design). Therefore, combining predictions does not substantially cancel out errors. Similarly, replica averaging exhibits strong diminishing returns. Averaging four replicas improves MUE by 6–11% compared to the single trial across methods. Notably, expanding from one to two replicas alone captures 50–73% of that total gain, while additional replicas contribute progressively less. Together, these results suggest that the dominant error source in current AFES workflow is systematic rather than statistical, and that investment in the force field quality, perturbation-network design, and structural input quality are likely to yield greater accuracy improvements than simply increasing replica count or combining methods.

The scope of this evaluation is restricted to 10 protein targets (8 from the JACS set and 2 case studies) and a relatively small set of ligand transformations, which limits the generalizability of the findings to broader chemical spaces. Each method also exhibits inherent sampling constraints that may affect convergence for complex transformations. Amber-TI and OpenMM-FEP rely on sufficient overlap between λ-windows, nonequilibrium switching in GROMACS-NETI introduces sensitivity to transition protocols, and BLaDE-MSLD requires careful parametrization and/or grouping to ensure stability and accuracy. Protein–ligand complex structural biases represent another source of uncertainty, as docking-derived initial poses and softcore parameter settings can influence the accuracy of predicted free energy changes, particularly for transformations involving ambiguous binding modes. Furthermore, the experimental data sets used in the two case studies exhibit a narrow range of ΔΔ*G*_bind_ values, which reduces the interpretability of correlation metrics as indicators of predictive performance and may obscure systematic errors. Additionally, while a minimal redundant graph was adopted in this study to maximize chemical coverage across diverse transformations within a practical computational budget, incorporating redundant cycles with cycle-closure constraints could reveal thermodynamic inconsistencies and further improve prediction accuracy. These limitations highlight the need for caution when extrapolating the results to systems with greater structural diversity or more pronounced energetic differences.

Future work should explore hybrid approaches that combine the strengths of these methods, such as integrating ligand grouping strategies for MSLD to improve sampling efficiency. Based on our internal testing, split-group configurations generally yield higher accuracy than single-group projects. However, aside from unavoidable edge cases such as p38 and PTP1B, these improvements are typically modest and, in some instances, may slightly decrease accuracy. To ensure higher numerical robustness, we recommend running at least 2 independent replicas to reduce statistical noise and enhance confidence in the final estimates. *MolCube-AFES* supports automatic ligand grouping for BLaDE-MSLD system generation, with the maximum number of substituents per site as 10 to ensure robust and broadly applicable performance.

Separately from sampling considerations, the availability of reliable structural priors is a critical factor for achieving accurate free energy predictions, particularly for complex protein–ligand systems. Recent advances in AI-based algorithms for protein–ligand complex structure generation^58^ provide a powerful means of supplying high-quality initial structures for downstream free energy calculations. The effectiveness of this approach is illustrated by our GR case study, in which protein–ligand complex structures generated using Boltz-2 were subsequently used to initialize AFES calculations, yielding high quantitative accuracy (MUE of around 1 kcal/mol) despite the narrow ΔΔ*G*_bind_ range of the data set. These results highlight the potential of integrating AI-derived structural models with physics-based free energy methods. Looking forward, further coupling such structure generation approaches with GPU-optimized free energy algorithms offers a promising pathway to improving robustness, accuracy, and scalability in drug discovery applications.

The current implementation of *MolCube-AFES* supports multiple protonation or tautomeric states and charge changing transformations, enabling broader applicability beyond strictly neutral, single state ligand perturbations. For charge-changing transformations, *MolCube-AFES* uses a symmetric net-charge boundary protocol to reduce the dominant PME finite-size artifact. Under periodic boundary conditions with PME electrostatics, the leading background charge artifact depends on the difference in the squared net charge between the two alchemical end points.^59^ Therefore, when the ligand charge changes by Δq, a static fractional spectator ion with charge −Δ*q*/2 is placed in the bulk solvent. For example, for a 0 to +1 ligand transformation, a −0.5e spectator ion gives end point net charges of −0.5e and +0.5e, making the squared net charge identical at the two end points. This construction cancels the leading Type I PME finite-size term by symmetry, following the charge-cancellation principle used in paired charge-mutation approaches.^60^ Thus, the dominant net-charge artifact is mitigated within the simulation setup without requiring an explicit a posteriori correction for this leading term. However, this protocol does not explicitly remove higher-order finite-size effects, such as residual solvent-polarization or ion-solvation terms, and the fractional spectator ion may locally perturb solvent structure. Therefore, although the leading Type I term is canceled by construction, careful placement of the spectator ion in the bulk solvent and sufficiently large solvation boxes remain necessary to reduce residual nonphysical interactions.

At the current stage, true scaffold modifications and grand-canonical water insertion/deletion protocols are not yet supported in *MolCube-AFES*. These features remain important targets for future development, and their implementation will further expand the applicability of *MolCube-AFES* to more challenging ΔΔ*G*_bind_ scenarios involving substantial ligand reorganization and water-mediated binding effects.

The present benchmark also reflects differences in the algorithmic and sampling capabilities available for each backend at the time of system preparation. Amber provides advanced features such as smoothstep softcore potentials^40^ and ACES-HREMD,^41,42^ which are designed to enhance alchemical stability and facilitate sampling across high-energy conformational barriers (e.g., ring flipping or significant torsional rearrangements).^61^ However, because our benchmark data set contained relatively few perturbations of this nature and utilized a fixed λ schedule across chemically diverse transitions, the application of these advanced options yielded negligible improvements in prediction accuracy (Figure S4). Consequently, these results should be interpreted within the context of the selected benchmark protocol rather than as a general limitation of Amber’s advanced capabilities. Future work will continue to align the available sampling options across the AFES backends to enable more controlled cross-method comparisons.

## Supplementary Material





## Data Availability

All input files used in this study are available at https://github.com/molcube/molcube_afes_rbfe.

## Notes

List of 10 transformations for 8 JACS set proteins (Table S1); ΔΔ*G*_bind_ accuracy metrics for the four methods (Table S2); BLaDE-MSLD system definitions (Table S3); ΔΔ*G*_bind_ differences between single-group and split-group MSLD (Table S4); ΔΔ*G*_bind_ about *Wang et al*. (Table S5 and Figure S1); single-GPU runtime per morph for thrombin transformations (Table S6); chemical structures of azaxanthene-based and TNI-97 compound series (Tables S7 and S8); ligand perturbation maps for the glucocorticoid receptor and HDAC6 systems (Figures S2 and S3); and MUE by target for the original, smoothstep-only, and smoothstep+ACES-HREMD protocols (Figure S4) (PDF)
Per-transformation ΔΔ*G*_bind_ values for all JACS benchmark, 3BQD, and 5EDU systems, including individual replicate (R1–R4) results and per-engine error metrics (XLSX)
