# Supplementary material for: Comparative Analysis of Relative Ligand Binding Free Energy Simulation Methods: Amber-TI, GROMACS-NETI, OpenMM-FEP, and BLaDE-MSLD
Source: J Chem Theory Comput. 2026 Oct 9;22(20):11056–76. doi: 10.1021/acs.jctc.6c01744 (PMC13718546; doi:10.1021/acs.jctc.6c01744)
Supplement: Supplementary file 1 [file ct6c01744_si_001.pdf]

Supporting Information

Comparative Analysis of Relative Ligand Binding Free Energy Simulation Methods: Amber-TI, GROMACS-NETI, OpenMM-FEP, and BLaDE-MSLD

Hajin Lee[1‡], Injung Kim[1‡], Sungjun Kim[1‡], Minsik Bae[1], Bomi Jeong[1], Seonghoon Kim[1], Sunhwan Jo[1], Jumin Lee[1*], and Wonpil Im[1,2*]

[1]MolCube Inc., Seoul 06704, Republic of Korea

[2]Department of Biological Sciences, Lehigh University, Bethlehem, PA 18015, USA

[‡]These authors contributed equally to this work

*Corresponding Authors: Jumin Lee (jumin@molcube.com) and Wonpil Im (wonpil@lehigh.edu)

Table S1. 10 transformations of 8 proteins in the JACS set.

Protein	RCSB ID	Ligand 1	Ligand 2
BACE	4DJW	CAT-4k	CAT-4o
		CAT-13o	CAT-17h
		CAT-17b	CAT-17e
		CAT-24	CAT-17i
		CAT-4m	CAT-4p
		CAT-4m	CAT-13j
		CAT-13d	CAT-13b
		CAT-13d	CAT-13h
		CAT-13g	CAT-17i
		CAT-13j	CAT-4o
Tyk2	4GIH	jmc_23	ejm_46
		jmc_23	ejm_55
		jmc_23	jmc_30
		ejm_47	ejm_31
		ejm_49	ejm_31
		ejm_55	ejm_54
		ejm_31	ejm_48
		ejm_31	ejm_45
		ejm_43	ejm_55
		ejm_44	ejm_42
CDK2	1H1Q	22	1h1r
		26	1oi9
		30	26
		31	32
		1h1r	1oi9
		1h1s	26
		1oi9	20
		1oiy	1h1q
		17	1h1q
		20	1h1q
MCL1	4HW3	35	33
		38	35
		42	51
		43	47
		26	57
		62	45
		28	35
		67	35
		67	53
		67	27

JNK1	2GMX	18634-1	18637-1
		18636-1	18624-1
		18637-1	18631-1
		18638-1	18634-1
		18659-1	18634-1
		18626-1	18634-1
		18626-1	18658-1
		18626-1	18659-1
		18626-1	18627-1
		18626-1	18628-1
p38	3FLY	p38a_2l	p38a_2j
		p38a_2l	p38a_2o
		p38a_2m	p38a_2j
		p38a_2m	p38a_2k
		p38a_2s	p38a_2l
		p38a_2v	p38a_3fln
		p38a_2v	p38a_3fmk
		p38a_3fly	p38a_2x
		p38a_3fly	p38a_3flq
		p38a_3flz	p38a_2c
thrombin	2ZFF	1a	5
		6e	6b
		1b	1a
		1b	3b
		1d	1a
		1d	1c
		1d	5
		3a	1b
		6a	1b
		6a	6b
PTP1B	2QBS	20667(2qbp)	23479
		23471	23468
		23477	23467
		23482	23479
		23484	23486
		20670(2qbs)	23477
		20670(2qbs)	23466
		23467	23475
		23467	23476
		23467	23466

Table S2. Comparison of $\Delta\Delta G_{bind}$ values across the four methods with experimental results.

Protein	Amber-TI				GROMACS-NETI				OpenMM-FEP				BLaDE-MSLD			
	RMSE[a]	MUE[a]	R^2	r_P	RMSE[a]	MUE[a]	R^2	r_P	RMSE[a]	MUE[a]	R^2	r_P	RMSE[a]	MUE[a]	R^2	r_P
**BACE**	1.16	0.88	0.46	0.68	1.12	0.86	0.45	0.67	1.14	0.98	0.14	0.37	0.88	0.61	0.60	0.78
**Tyk2**	1.05	0.81	0.51	0.71	1.09	0.85	0.42	0.64	1.14	0.85	0.34	0.58	1.05	0.89	0.54	0.73
**CDK2**	1.13	0.79	0.44	0.66	1.04	0.75	0.48	0.69	1.07	0.84	0.47	0.68	1.27	0.96	0.28	0.53
**MCL1**	2.30	1.72	0.26	0.51	2.04	1.64	0.29	0.54	2.48	1.84	0.27	0.52	2.37	1.99	0.16	0.40
**JNK1**	1.15	0.97	0.26	0.51	1.30	1.04	0.18	0.43	1.32	1.13	0.14	0.38	1.07	0.80	0.11	-0.33
**p38**	1.58	1.23	0.51	0.71	1.15	0.99	0.66	0.81	1.45	1.08	0.53	0.73	1.06	1.30	0.43	0.65
**thrombin**	0.61	0.45	0.36	0.60	0.73	0.56	0.39	0.63	0.62	0.50	0.48	0.70	0.98	0.73	0.39	0.63
**PTP1B**	2.02	1.43	0.01	0.12	1.76	1.25	0.20	0.45	2.32	1.62	0.01	-0.10	1.84	1.26	0.13	0.36

[a]The unit is kcal/mol.

Table S3. Systems used in BLaDE-MSLD. The group id, number of ligands, number of sites, and number of substituents are listed.

Protein	Group ID	# ligands	# Sites	# Subs
BACE		15	2	7 × 9
Tyk2		13	1	13
CDK2		12	2	6 × 7
MCL1		15	2	12 × 7
JNK1		11	4	3 × 4 × 4 × 4
p38	p38-1	14	3	5 × 6 × 6
	p38-2a	6	1	6
	p38-2b	4	1	4
	p38-2c	6	1	6
thrombin		10	2	4 × 7
PTP1B	PTP1B-1	13	2	3 × 11
	PTP1B-2a	5	2	3 × 3
	PTP1B-2b	9	1	9

Table S4. $\Delta\Delta G_{bind}$ prediction difference in BLaDE-MSLD using 1 group and split groups.

Protein	Ligand 1	Ligand 2	$\Delta\Delta G_{1group}$	$\Delta\Delta G_{groups}$	exp.
p38	p38a_2l	p38a_2j	-1.82	-0.94	2.18
	p38a_2s	p38a_2l	0.28	-0.42	-1.15
	p38a_3fly	p38a_2x	0.13	-0.17	1.19
	p38a_3flz	p38a_2c	0.06	-1.54	-0.97
	p38a_2l	p38a_2o	0.76	0.88	1.77
	p38a_2m	p38a_2k	1.78	2.04	0.41
	p38a_3fly	p38a_3flq	0.13	-0.51	1.49
	p38a_2m	p38a_2j	-1.51	-0.63	0.88
	p38a_2v	p38a_3fln	-1.49	-1.64	-1.91
	p38a_2v	p38a_3fmk	-1.54	-2.80	-2.86
	MUE[a]		1.54	1.22	
	r_p		0.23	0.60	
PTP1B	23467	23476	-6.30	-0.73	-2.07
	23471	23468	-5.50	0.08	0
	20670(2qbs)	23477	-1.06	0.23	0.23
	23477	23467	7.09	0.20	1.51
	23467	23466	4.63	0.05	-0.51
	23482	23479	33.19	-0.22	0.86
	20670(2qbs)	23466	10.66	0.48	1.24
	23467	23475	-6.21	-0.48	-1.38
	23484	23486	1.08	-0.51	-4.72
	20667(2qbp)	23479	-45.28	-0.13	2.28
	MUE[a]		12.17	1.27	
	r_p		-0.11	0.67	

[a]The unit is kcal/mol.

Table S5. Comparison of $\Delta\Delta G_{bind}$ values from the Wang et al. with experimental results.

	RMSE[a]	MUE[a]	Correlation R^2	r_P	Acc ±1	Acc ±2
**Wang et al.**	1.27	0.99	0.35	0.65	0.58	0.89

[a]The unit is kcal/mol.

Table S6. Single-GPU runtime (hours) per morph for 10 thrombin alchemical transformations.

	Amber-TI			GROMACS-NETI			OpenMM-FEP		
	**Complex**	**Ligand**	**Total**	**Complex**	**Ligand**	**Total**	**Complex**	**Ligand**	**Total**
1a-5	9.26	4.22	13.48	9.33	3.46	12.78	10.63	2.73	13.36
6e-6b	9.41	4.06	13.47	9.20	3.24	12.45	11.12	3.08	14.20
1b-1a	9.23	4.07	13.30	8.63	2.10	10.74	10.43	2.73	13.16
1b-3b	9.44	4.18	13.62	9.48	3.52	13.00	10.78	2.92	13.70
1d-1a	9.23	4.33	13.55	9.59	3.05	12.64	10.78	2.75	13.53
1d-1c	9.40	4.08	13.47	8.65	2.02	10.67	10.60	2.77	13.37
1d-5	9.11	4.35	13.45	9.02	3.10	12.13	10.58	2.72	13.30
3a-1b	9.63	4.22	13.86	9.22	2.74	11.96	10.45	2.98	13.43
6a-1b	9.53	4.17	13.71	8.45	2.17	10.63	10.53	2.93	13.46
6a-6b	9.43	4.17	13.6	8.70	2.63	11.34	10.88	3.05	13.93
Avg.			13.55			11.83			13.54

Table S7. Chemical structures of azaxanthene-based compounds used for AFES calculations.

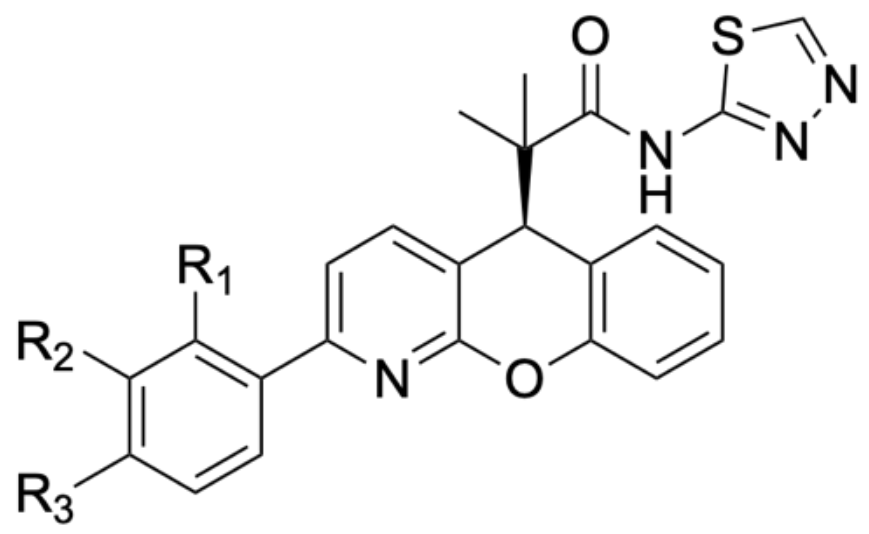

Compounds	Substitution Site	Functional Group
**14**	-	-
**15**	R1	Me
**16**	R2	Me
**17**	R3	Me
**19**	R3	OMe
**20**	R3	Et
**21**	R3	*n*Pr
**22**	R3	O^{i}Pr
**23**	R3	OH
**24**	R3	NMe_2
**26**	R3	Ac
**27**	R2	F
	R3	O^{i}Pr
**28**	R2	F
	R3	Ac

Table S8. Chemical structures of TNI-97 compound series used for AFES calculations.

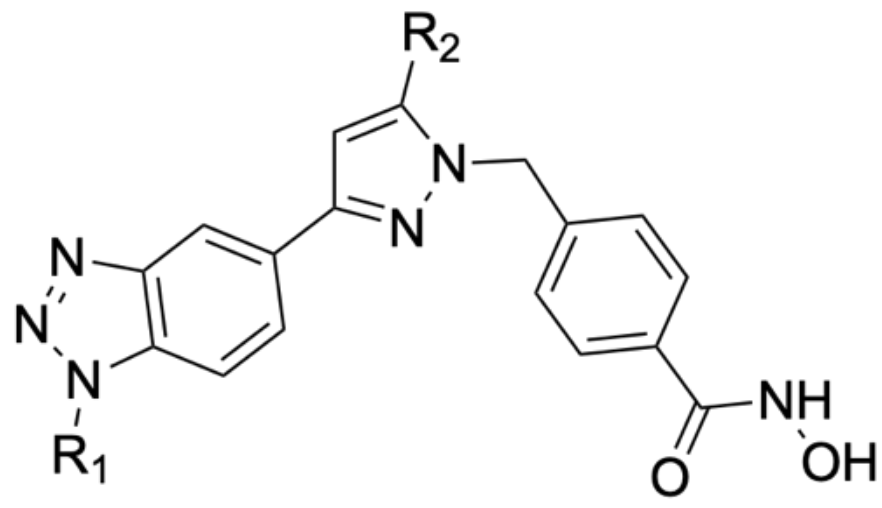

Compounds	R1	R2
**30a**	$-CH_3$	CF_3
**30b**		Ph
**30c**		$4-CF_3Ph$
**30d**		$2-CH_3Ph$
**30e**		$3-CH_3Ph$
**30f**		$4-CH_3Ph$
**30g**	$-C_2C_5$	CF_3
**30h**	$-C_2C_5OCH_3$	CF_3
**30i**	$-CH(CH_2)_2$	CF_3
**30j**		Ph
**30k**		4-F-Ph
**30l**		$4-CF_3Ph$
**30m**		Bn
**30n**	-Bn	CF_3
**30o**		$4-CF_3Ph$
**30p**		$2-CH_3Ph$
**30q**		$3-CH_3Ph$
**30r**		$4-CH_3Ph$

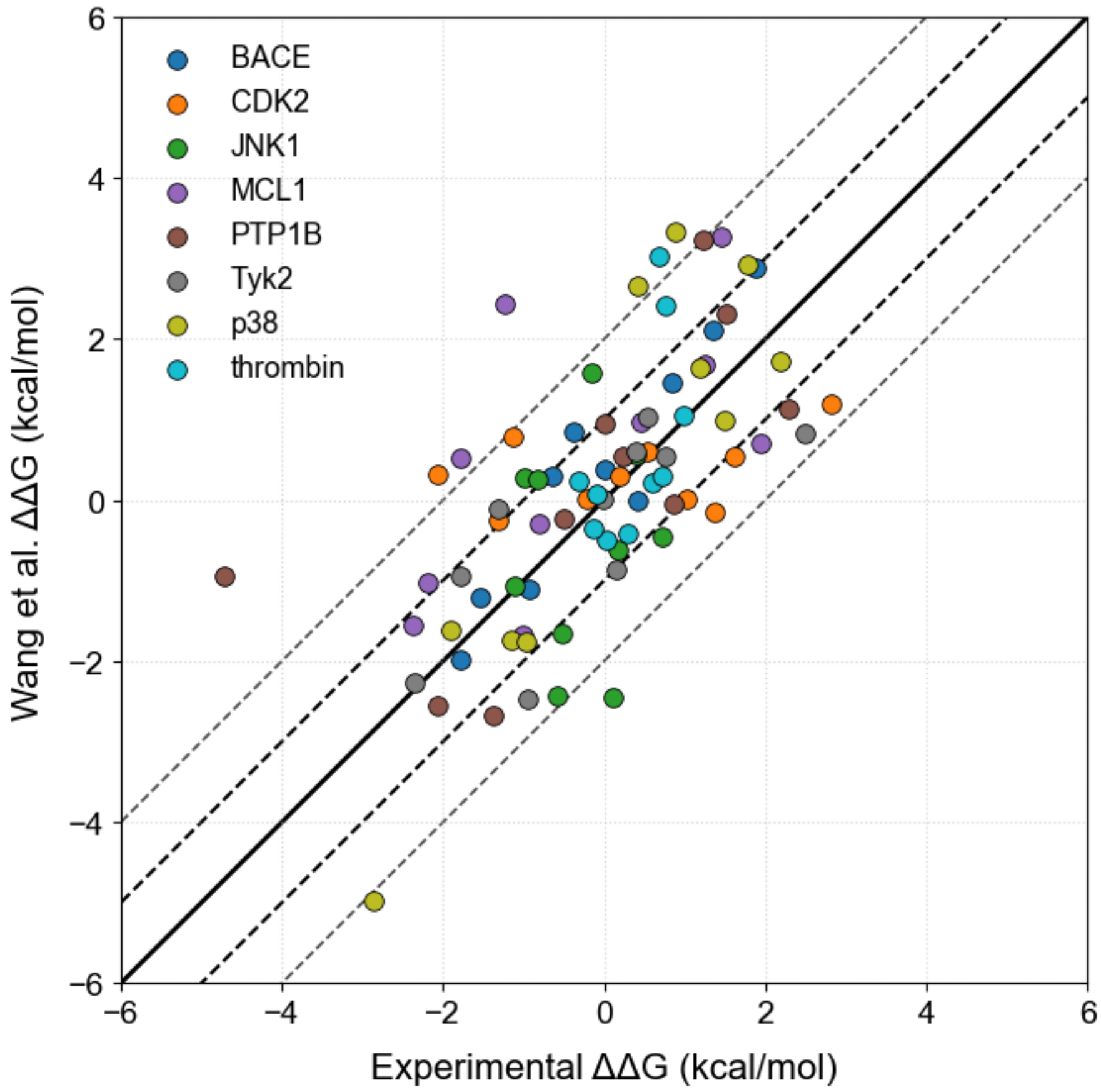

Figure S1. $\Delta\Delta G_{bind}$ prediction-experiment reported by Wang et al..

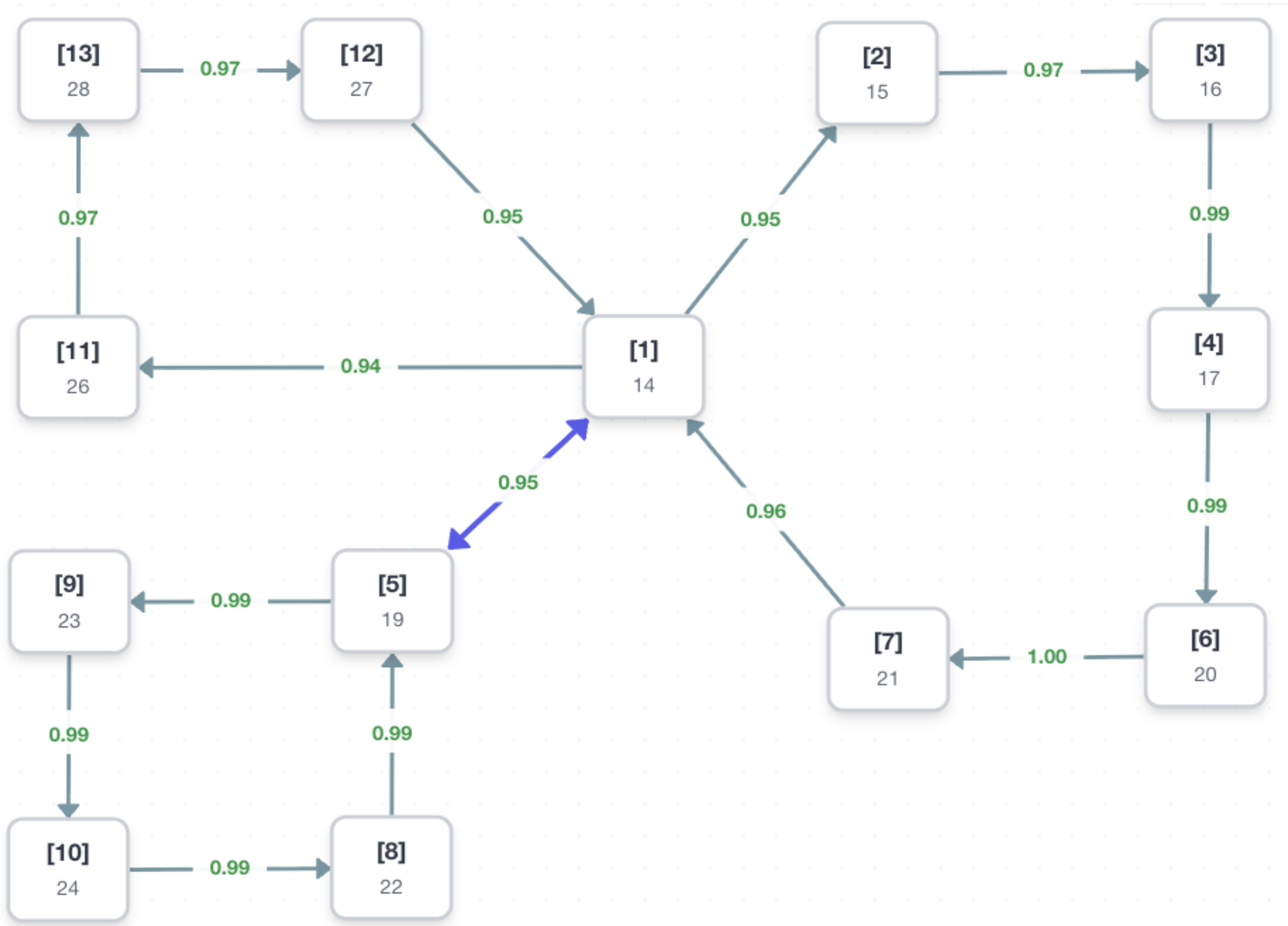

Figure S2. Ligand perturbation map for the glucocorticoid receptor (3BQD) system. Each node represents a ligand, labeled with its compound name (in **Table S7**), and arrows indicate the direction of the relative free energy perturbations. The numerical value displayed near each arrow represents the similarity score between the connected nodes. Multiple closed cycles were generated by connecting ligands with similar functional groups or heteroatoms to ensure high phase-space overlap. The compound numbering starts from [1] on the map corresponding to compound 14.

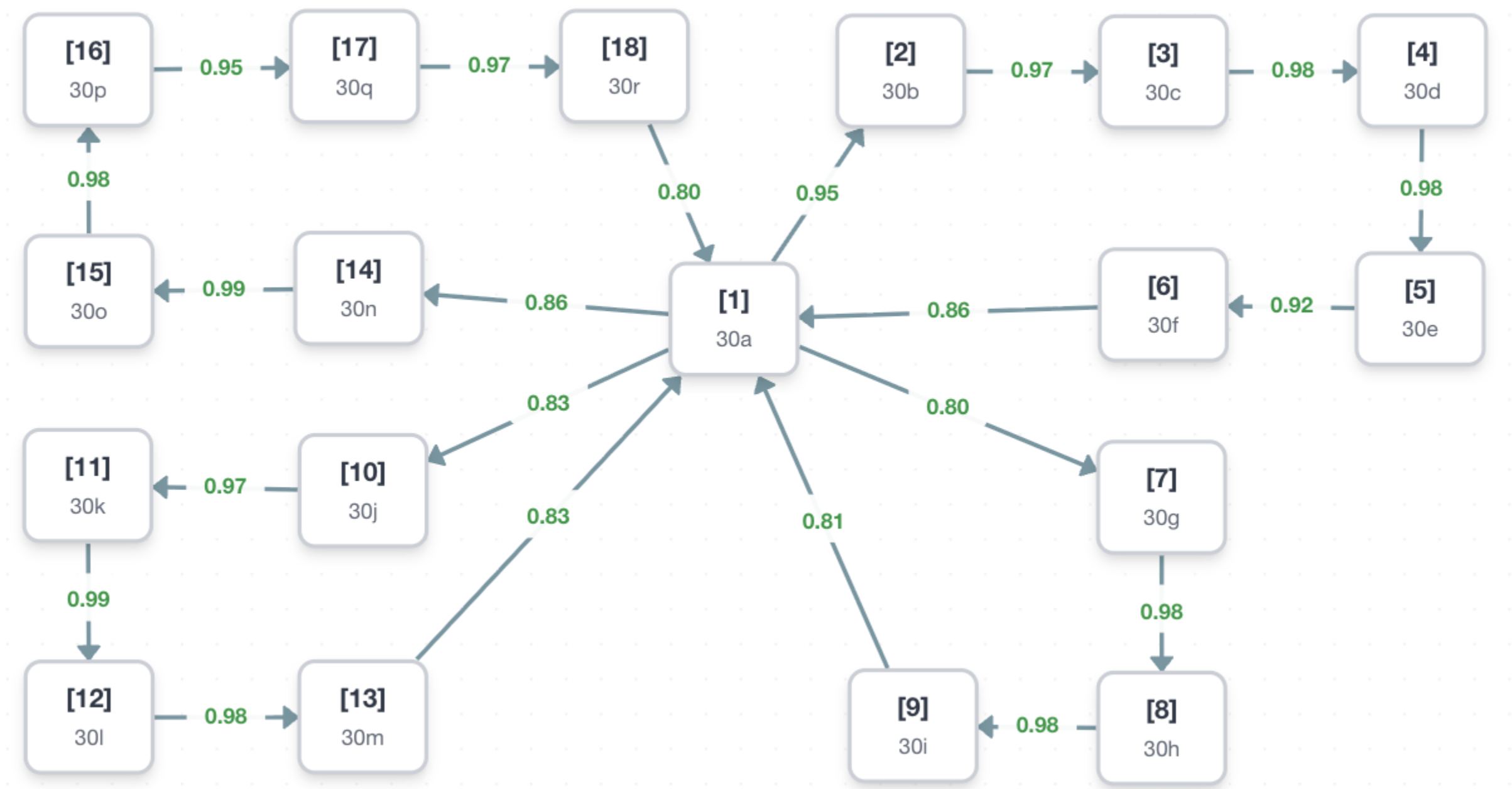

Figure S3. Ligand perturbation map for the HDAC6 inhibitor (5EDU) system. Each node represents a ligand, labeled with its compound name (in **Table S8**), and arrows indicate the direction of the relative free energy perturbation. The numerical value displayed near each arrow represents the similarity score between the connected nodes. Multiple closed cycles were constructed between ligands bearing diverse R1 and R2 substituents to ensure high phase-space overlap. The compound numbering starts from [1] on the map corresponding to compound 30a.

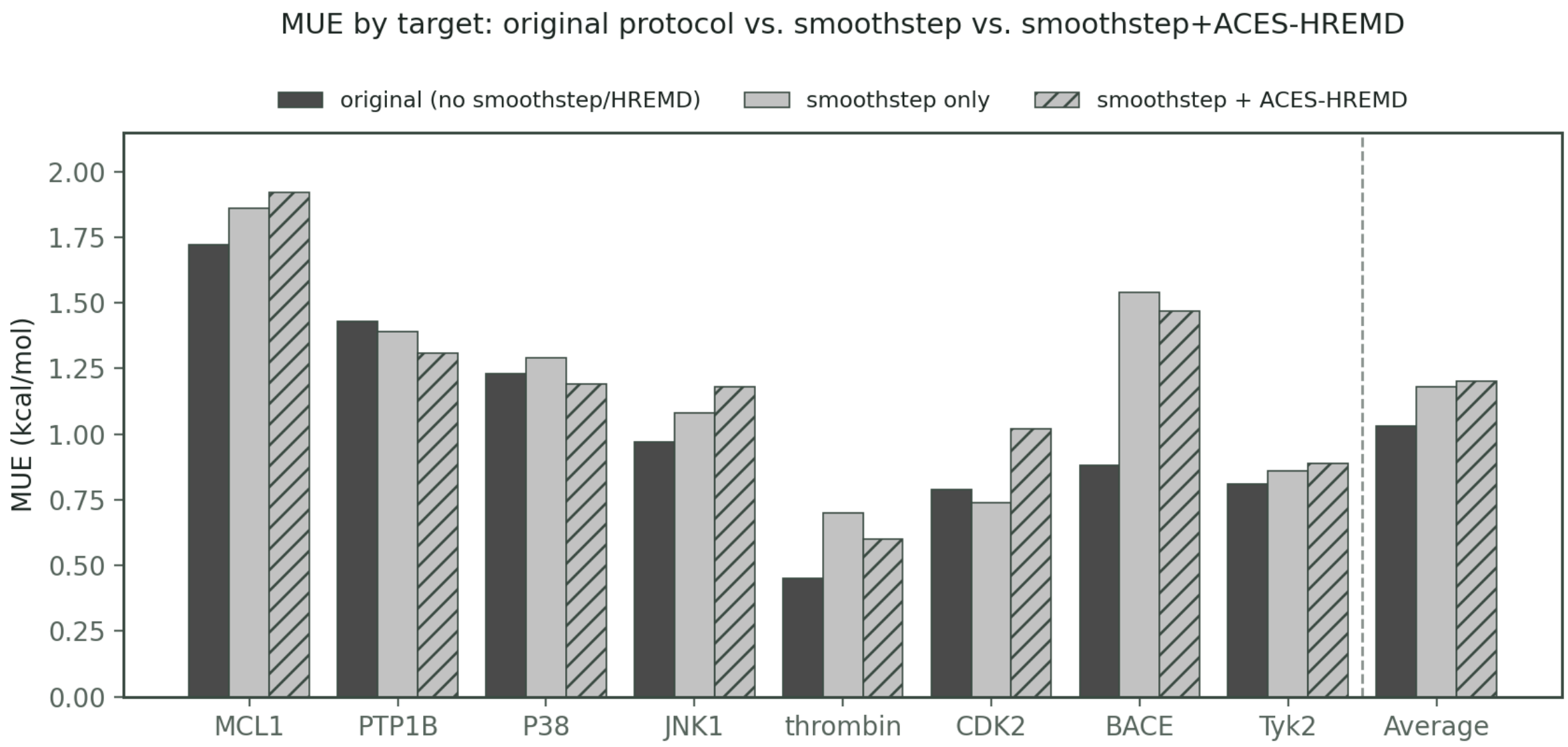

Figure S4. MUE by target for the original protocol, smoothstep only, and smoothstep+ACES-HREMD. MUEs for the eight benchmark proteins and the mean across all eight proteins are shown. Values for the original protocol are averages over four independent trials, whereas those for smoothstep only and smoothstep+ACES-HREMD are from a single trial each. Accordingly, apparent differences among the protocols should be interpreted in light of the run-to-run variability inherent in alchemical free-energy calculations and the unequal numbers of trials.
